# Enhancing mitophagy by ligustilide through BNIP3-LC3 interaction attenuates oxidative stress-induced neuronal apoptosis in spinal cord injury

**DOI:** 10.7150/ijbs.98051

**Published:** 2024-08-12

**Authors:** Hui Yao, Chaoyang Cai, Weijun Huang, Caizhen Zhong, Tianlun Zhao, Jiawei Di, Juliang Tang, Depeng Wu, Mao Pang, Lei He, Limin Rong, Bin Liu

**Affiliations:** 1 Department of Orthopaedics, The Third Affiliated Hospital of Sun Yat-sen University, Guangzhou, PR China; 2 Department of Spine Surgery, The Third Affiliated Hospital of Sun Yat-sen University, Guangzhou, PR China; 3 Guangdong Provincial Center for Quality Control of Minimally Invasive Spine Surgery, Guangzhou, PR China; 4 Guangdong Provincial Center for Engineering and Technology Research of Minimally Invasive Spine Surgery, Guangzhou, PR China; 5 Department of Gastroenterology and Rheumatology, The Third Affiliated Hospital of Sun Yat-sen University, Guangzhou, PR China

**Keywords:** ligustilide, mitochondrial autophagy, neuronal apoptosis, non-ubiquitination process, ROS, spinal cord injury

## Abstract

Mitophagy selectively eliminates damaged or dysfunctional mitochondria, playing a crucial role in maintaining mitochondrial quality control. However, it remains unclear whether mitophagy can be fully activated and how it evolves after SCI. Our RNA-seq analysis of animal samples from sham and 1, 3, 5, and 7 days post-SCI indicated that mitophagy was indeed inhibited during the acute and subacute early stages. *In vitro* experiments showed that this inhibition was closely related to excessive production of reactive oxygen species (ROS) and the downregulation of BNIP3. Excessive ROS led to the blockage of mitophagy flux, accompanied by further mitochondrial dysfunction and increased neuronal apoptosis. Fortunately, ligustilide (LIG) was found to have the ability to reverse the oxidative stress-induced downregulation of BNIP3 and enhance mitophagy through BNIP3-LC3 interaction, alleviating mitochondrial dysfunction and ultimately reducing neuronal apoptosis. Further animal experiments demonstrated that LIG alleviated oxidative stress and mitophagy inhibition, rescued neuronal apoptosis, and promoted tissue repair, ultimately leading to improved motor function. In summary, this study elucidated the state of mitophagy inhibition following SCI and its potential mechanisms, and confirmed the effects of LIG-enhanced mitophagy through BNIP3-LC3, providing new therapeutic targets and strategies for repairing SCI.

## Introduction

Spinal cord injury (SCI) is a devastating condition that always leads to irreversible neurological deficits, significantly impairing sensorimotor function and even resulting in death 1. SCI triggers a series of progressive pathophysiological changes, encompassing both primary and secondary damage. The primary damage occurs within 2 hours of the initial forces, such as compression, contusion, transection, and shearing, which are so sudden that there is no time for a response. Following the primary injury, the secondary damage, categorized into acute, subacute, and chronic stages, can persist for days, weeks, or even years. Several molecular and metabolic events are involved in the acute and subacute stages, including elevated glutamate levels, Ca^2+^ overload, oxidative stress, and mitochondrial dysfunction, ultimately leading to neuronal apoptosis 2-4. Neuronal apoptosis is a critical pathological event contributing to neurological deficits 5. Numerous studies have been conducted to address neuronal apoptosis 6-9, but effective strategies to reverse neuronal cell death and restore neurological function are still lacking.

The resulting ischemic damage and cellular dysregulation after SCI lead to the accumulation of free radicals such as reactive oxygen species (ROS), including nonradical hydrogen peroxide (H_2_O_2_), hydroxyl radical (·OH), and superoxide radical anion (O2-) 4, 10, 11. Excessive ROS generation exceeds the scavenging capacity of the antioxidant system, causing damage to tissues, cells, and biological macromolecules, and exacerbating SCI 9, 11, 12. Mitochondria, considered as the "powerhouse" of the cell, play a vital role in maintaining cellular homeostasis related to adenosine triphosphate (ATP) production, regulation of reactive oxygen species (ROS), and apoptosis 13. Mitochondria are known to be the primary source of endogenous ROS due to their respiratory chain 14. Limited cellular ROS levels can be beneficial for normal physiological functions, while excessive ROS accumulation leads to mitochondrial damage, manifested as collapse of mitochondrial membrane potential (MMP), decrease in ATP levels, translocation of dynamin-related protein 1 (DRP-1) into the mitochondria, and upregulation of proteins involved in the mitochondria-related apoptosis pathway 4, 15-18. Defective mitochondria can further be toxic by generating more ROS, consuming ATP through the reversal of ATP synthase, and interfering with a host of other metabolic processes 19, leading to a vicious cycle. Therefore, restoring mitochondrial function or appropriately clearing defective mitochondria may contribute to SCI repair.

Autophagy is an intracellular lysosome-dependent pathway for degrading organelles or pathological proteins and plays a crucial role in maintaining cellular homeostasis 20-22. Mitophagy is a specialized form of autophagy that selectively clears damaged or dysfunctional mitochondria through autophagic machinery and functions to maintain mitochondrial quality control and homeostasis 13,23-25. Reports on the spatial and temporal patterns of autophagy activation after traumatic SCI have been contradictory 26,27, and different injury severity in various animal models has led to differential activation of autophagy 28. Strategies involving both activation and inhibition of autophagy for targeting SCI have been reported, making it complex to choose the right approach. Studies have shown that there is an inherent need in the spinal cord to activate enough autophagy to promote cell survival after the primary traumatic injury 27, 29-33. However, the pathophysiological events involved in the secondary injury may disrupt the autophagy flux, such as excessive ROS generation, which causes oxidative damage to the lysosomal membrane and subsequently blocks the fusion of autophagosomes and lysosomes 16, 27, 29, 34. Therefore, the eventual evolution of autophagy in traumatic SCI remains unclear. Mitophagy is neuroprotective due to its ability to reduce the production of ROS by clearing dysfunctional mitochondria 35. Previous studies have shown that the blockage of mitophagy flux could lead to cell death 36,37,38. Whether excessive ROS in traumatic SCI block the mitophagy flux and lead to neuronal cell death eventually has been poorly explored. We speculate that ROS-induced defective mitophagy flux exacerbates oxidative damage to neurons and provides an overabundant apoptotic stimulus far above their threshold.

Ligustilide (3-butylidene-4,5-dihydroisodenzofuranone; LIG) is one of the main active ingredients isolated from traditional Umbelliferae family medicine, such as Ligusticum chuanxiong Hort and Angelica sinensis. It has been well documented that LIG is neuroprotective due to its ability to cross the blood-brain barrier 39,40, its anti-inflammatory effect 41,42, and its antioxidative and antiapoptotic effects 43,44. Additionally, studies have reported that LIG promoted mitochondrial fission in ischemic stroke injury 45 and activated mitophagy in hippocampal neuronal injury 46. However, the protective role of LIG in SCI has been poorly explored. We hypothesize that LIG restores ROS-induced defective mitophagy flux, attenuates neuronal cell death, and alleviates SCI impairments. Besides, the corresponding mechanisms were also explored in this study. We established an *in vivo* SCI contusion mice model and an *in vitro* oxidative stress model to demonstrate the neuroprotective effects of LIG against ROS-exacerbated SCI and to reveal its underlying mechanisms, potentially providing a new strategy to overcome devastating SCI.

## Material and methods

### Animals and groups

The animal experiments were conducted in two batches. The first batch consisted of the SHAM group and the SCI 1, 3, 5, and 7-day groups, totaling 58 animals. The second batch comprised four groups: SHAM, SCI+Vehicle, SCI+LIG20, and SCI+LIG50, totaling 76 animals. All animals were purchased from the Guangdong Medical Laboratory Animal Center and were 8-week-old female C57BL/6 mice. They were housed in a specific pathogen-free facility at the Experimental Animal Center of South China Agricultural University. All surgical procedures and postoperative care protocols were approved by the Animal Care and Use Committee of South China Agricultural University (approval number: 2023D064).

The surgical procedures were briefly described as follows: Mice were anesthetized through an intraperitoneal injection of sodium pentobarbital (40 mg/kg). A laminectomy was performed at the T9-10 level, and the exposed spinal cord was lesioned using the Infinite Horizon Impactor (Precision Systems and Instrumentation, LLC) to induce a moderate (70 kD) contusion. In the SHAM group, only a laminectomy was carried out without contusion injury. LIG (purity 98%, L886269, Macklin, Shanghai, China) was dissolved in 3% Tween 80 (PH1516, Phygene, Fuzhou, China). SCI mice received daily intraperitoneal injections of LIG 20mg/kg/d 45,46, 50mg/kg/d 42, and an equal dose of 3% Tween 80 for one week after operation were respectively designated as SCI+LIG20, SCI+LIG50 and SCI+Vehicle group.

### Cell culture and drug application

Rat-derived PC12 cells were cultured in DMEM (Gibco, USA) supplemented with 10% fetal bovine serum (FBS, Gibco, USA) and 1% penicillin/streptomycin (Life Technologies, USA). Tert-butyl hydroperoxide solution (TBHP) was purchased from Sigma-Aldrich (458139; USA), and the optimal working concentration was determined to be 50 μM for 6 hours to simulate excessive ROS damage to neurons in SCI (Fig. S1A). LIG (purity 98%, L886269, Macklin, Shanghai, China), dissolved in Dimethyl Sulfoxide (DMSO) with the final concentration less than 0.1% (vol/vol), showed its optimal effective concentration at 20 μM and 50 μM for 24h (Fig. S1B). Urolithin A (UA, purity≥98%, U884401, Macklin, Shanghai, China) was used as a mitophagy agonist for PC12 cells at 5 μM 47,48 for 24h. Mdivi-1, a well-known mitophagy inhibitor, was purchased from MCE (HY-15886, New Jersey, USA) and was applied to inhibit mitophagy of PC12 cells at 25 μM 45 for 6 h.

### siRNA transfection

The siRNA sequence for silence BCL2 interacting protein 3 (BNIP3) was synthesized by Obio Technology (Shanghai, China). PC12 cells were transfected according to the manufacturer's instructions with the aid of Lipofectamine™ 2000 Transfection Reagent (11668030, Invitrogen, California, USA). Three alternative sequences and the control sequence are as shown in Table S1. After 24 h of siRNA transfection, the best sequence for the tests was chosen by qRT-PCR and finally confirmed with western blot.

### CCK-8 assay

Cells were seeded in 96-well plates at a density of 5×10^3^ cells in 100 μL DMEM per well and incubated in a cell culture incubator for 24 hours. After medication, 10 μL of CCK-8 solution (CK04; DOJINDO, Japan) was added to each well, and the cells were further incubated for 1-4 hours. Subsequently, the Optical Density (OD) values at 450 nm were measured using a microplate reader (MK3; Thermo; USA), and cell viability was calculated using the following equation: Cell viability (%) = [OD (sample) - OD (blank)] / [OD (control) - OD (blank)] × 100.

### RNA extraction and qRT-PCR

Total RNA was extracted from PC12 cells using the total RNA extraction kit (Omega, USA) and from spinal cord samples using Trizol (Thermo Scientific, USA) following the manufacturer's instructions. Subsequently, single-stranded cDNA was synthesized from the extracted RNA using the PrimeScript RT Master Mix (Takara Bio, Japan). The mRNA expression levels of the target genes were assessed using qRT-PCR, which was performed with the LightCycler 480 SYBR Green I Master on the LightCycler 480 instrument (Roche, Germany). The specific primers for the target genes are listed in Table S2.

### Protein extraction and western Blot

Mitochondrial and cytoplasmic proteins were isolated using the extraction kit (BB-3171, BestBio, Shanghai, China) following the manufacturer's instructions. Total proteins from the cells and fresh spinal cord tissue were isolated using RIPA lysis buffer (Beyotime, China) containing Protease and Phosphatase Inhibitor Cocktail (Thermo, USA). The protein concentration was determined using the Pierce BCA Protein Assay Kit (Thermo, USA). Subsequently, 40 μg of protein extracts were loaded onto 8% or 12% sodium dodecyl sulfate polyacrylamide gel electrophoresis gels based on the molecular weight of the target proteins and then transferred onto a polyvinylidene fluoride membrane (Millipore, USA). The membrane was blocked with 5% skim milk for 1 hour, followed by an overnight incubation with primary antibodies at 4 °C. The primary antibodies used in this study were listed as follows: Bcl2 (1:1000; ab59348; Abcam), Bax (1:1000; ab32503; Abcam), Cleaved-Caspase 3 (1:000; 9661; Cell Signaling Technology), Cleaved-PARP (1:000; 95427; Cell Signaling Technology), Cyto C (1:5000; ab133504; Abcam), Lamp2(1:1000, ab199946, Abcam), Tomm20 (1:5000; ab56783; Abcam), P62 (1:1000; 5114; Cell Signaling Technology), LC3A/B(1:1000; 12741; Cell Signaling Technology), BNIP3 (1:1000, k004134p, Solarbio), β-actin (1:5000, GTX109639, GeneTex), α-tubulin (1:2000, 66031, Proteintech), VDAC (1:1000, ML122111, PTMA). After thoroughly washing the PVDF bands, they were incubated for 1 hour at room temperature with the following secondary antibodies: mouse IgG HRP-linked antibody (1:2000; ab205719; Abcam) and rabbit IgG HRP-linked antibody (1:2000; 7074; Cell Signaling Technology). The protein bands were then immersed in ECL (A38556; Thermo, USA) and captured using an automatic chemiluminescence system (Tanon, China). The intensity (pixels/band) was analyzed using the ImageJ densitometry analysis software.

### TUNEL assay

The spinal cord sections were deparaffinized with xylene and dehydrated with different concentrations of ethanol. After immunofluorescence staining with NeuN, the tissue sections were cultured with Tunel staining solution (C1088, Beyotime, China) in a dark and humid environment at 37 °C, for 2 hours. PC12 cells, preliminarily fixed with 4% paraformaldehyde, were subjected to wash with PBS and then cultured with Tunel staining solution (C1088, Beyotime, China) for 1 h. Finally, both were stained with Hoechst 33258 (Beyotime, China) at room temperature for 15 minutes. Images were captured with a fluorescence microscope (200×magnification; Nikon, Japan), and three randomly chosen fields of view were selected for each sample.

### Flow cytometry

5 μl Annexin V and Propidium Iodide (PI) (BD556547; BD Biosciences, USA) were both added to cell suspension. After incubation at 37 °C for 10 min, labeled cells were quantified using a flow cytometry (Beckman Coulter, USA) and at least 10,000 events per sample were recorded.

### Measurements of cellular ROS

To detect the total intracellular ROS level, cells were stained with dihydroethidium (DHE; CA1420; Solarbio, China), dissolved in serum-free medium (1:1000 dilution) to a final concentration of 5 μmol/L, for 30 min at 37℃.

MitoSOX probe (M36008; Thermo, USA) was utilized to detect mitochondria-specific ROS. After dilution at a ratio of 1:1000, it was added to the cell culture medium. MitoTracker Green probe (C1048, Beyotime, Shanghai, China), diluted at a ratio of 1:5000, was used to label mitochondria. After being washed 3 times with PBS, cells were co-incubated with both MitoSOX and MitoTracker Green for 30 minutes at 37℃. Fluorescence images were obtained with a fluorescence microscope (200× magnification; Nikon, Japan).

### Measurements of MMP

The MMP of cells was measured using JC-1 staining assay kit (HY-K0601; Mid-Columbia Engineering, USA). After treatment, 2 μM JC-1 staining solution was added to the medium and incubated with cells for 30 min at 37℃. After wash with PBS, the fluorescence changes were detected with a fluorescence microscope (200× magnification; Nikon, Japan).

### Immunofluorescence

After treatment, cells, cultured on confocal dishes at the appropriate density, were fixed with 4% PFA for 15 min and then were permeabilized with 0.1% Triton X-100 for 5 min. After blocking with 3% BSA for 1 h, cells were incubated with primary antibodies LC3B (1:200; 12741; Cell Signaling Technology), Tomm20 (1:500; ab56783; Abcam), BNIP3 (1:200, k004134p, Solarbio) at 4℃ overnight. Cells were washed with PBS three times and incubated with fluorescent secondary antibody including goat anti-mouse IgG H&L (Alexa Fluor® 488, ab6785, Abcam, USA), goat anti-mouse IgG H&L (Alexa Fluor® 594, A32727, Thermo, USA), goat anti-rabbit IgG H&L (Alexa Fluor® 488, A11070, Thermo, USA), and goat anti-rabbit IgG H&L (Alexa Fluor® 594, #8889, CST, USA) for 1 h. Nuclei were stained with Hoechst 33258 (Beyotime, China) at room temperature for 15 min. The fluorescence changes were detected with a SP8 lightning confocal microscope (630× magnification; Leica, Germany).

The spinal cord sections were deparaffinized and dehydrated using xylene and graded alcohols, followed by antigen retrieval using citrate antigen retrieval solution and cell membrane permeabilization using Triton X-100. Subsequently, the sections were blocked with 10% goat serum in PBS for 1 h at room temperature and then incubated with the following antibodies overnight at 4°C: LC3B (1:200; 12741; Cell Signaling Technology), Tomm20 (1:500; ab56783; Abcam), GFAP (1:1000; ab4674; Abcam ), NF200 (1:500; ab19857; Abcam), NeuN (1:500; ab177487; Abcam). Following this, secondary antibodies including goat anti-mouse IgG H&L (Alexa Fluor® 488, ab6785, Abcam, USA), goat anti-mouse IgG H&L (Alexa Fluor® 594, A32727, Thermo, USA), goat anti-rabbit IgG H&L (Alexa Fluor® 488, A11070, Thermo, USA), and goat anti-rabbit IgG H&L (Alexa Fluor® 594, #8889, CST, USA) were applied for 2 hours at room temperature. Nuclei were stained with Hoechst 33258 (Beyotime, China) at room temperature for 15 min. Images were captured using a SP8 lightning confocal microscope (630× magnification; Leica, Germany).

### Transmission electron microscopy (TEM)

Following overnight fixation in 2.5 % (w/v) glutaraldehyde and post-fixation in 2 % (w/v) osmium tetroxide, PC12 cells were stained with 2 % (w/v) uranyl acetate and dehydrated in acetone. Subsequently, semithin sectioning and toluidine blue staining were performed, and images were captured using a Hitachi transmission electron microscope.

### RNA sequencing

Mice for RNA Sequencing were subjected to sham and SCI operation. Spinal cord samples were collected as five groups: sham, and 1,3,5,7 days after SCI, 3 samples per group. Total RNA was extracted from the tissues using Trizol (Invitrogen, Carlsbad, CA, USA) according to manual instruction, then qualified and quantified using a Nano Drop and Agilent 2100 bioanalyzer (Thermo Fisher Scientific, MA, USA). RNA sequencing was performed on MGIseq2000 platform (BGI-Shenzhen, China). Genes with an adjusted p (q value)<0.05 and absolute fold change>2 were considered as differentially expressed genes (DEGs). Kyoto Encyclopedia of Genes and Genomes (KEGG), Gene Ontology (GO) enrichment and Hierarchical Clustering analysis were performed via the R package (v 3.5.1). All RNA-sequencing original data has been uploaded to Sequence Read Archive (SRA) database with the number PRJNA753793 (https://www.ncbi.nlm.nih.gov/sra/?term=PRJNA753793).

### Measurements of oxidative stress biomarkers

7 days after surgical procedure and drug application, some mice were euthanized by carbon dioxide suffocation. Spinal cord tissues were collected and the corresponding homogenate was prepared for the subsequent measurements. Glutathione (GSH) content (BC1175, Solarbio, Shanghai, China), Superoxide dismutase (SOD) activity (S0101S, Beyotime, Shanghai, China) and Malondialdehyde (MDA) content (S0131S, Beyotime, Shanghai, China) were determined according to the manufacturer's instructions.

### HE and Nissl staining

The spinal cord tissue fixed with paraformaldehyde was embedded in paraffin, sliced, deparaffinized in xylene and dehydrated in anhydrous ethanol, then subjected to HE and Nissl staining. The observation and photography were conducted under a light microscope (Nikon, Tokyo, Japan).

### Motor Evoked Potential (MEP) analysis

Four weeks post-surgery, MEP analysis was conducted on mice from different groups to assess the recovery of nerve conduction. The mice were anesthetized with isoflurane and secured in a stereotaxic apparatus. The skin on the dorsal side of the hind thigh of the mice was incised, and the muscles were separated to locate the sciatic nerve, which was then secured to a protective electrode and kept moist with physiological saline. The skin was incised at the midline, 1 mm to the left of the sagittal suture and 1 mm posterior to the coronal suture. A cranial drill was used to expose the dura mater, and the stimulating electrode was placed on the dura mater. The recording electrode was placed in the contralateral gastrocnemius muscle, with the tail grounded. Pulse electrical stimulation (duration: 5 ms; frequency: 1 Hz; voltage: 5 V) was applied to evoke MEP, and the waveforms were recorded. Differences in nerve conduction function recovery were analyzed through the latency and amplitude of the P wave.

### Footprint analysis

Four weeks after surgery, footprint analysis was conducted on the mice. Mice from different groups were trained to traverse a runway covered with white paper and their dark-seeking behavior was induced using a dark box to motivate them to reach the endpoint. Subsequently, ink was evenly applied to the hind paws of the mice, and their paw prints were recorded as they traversed the runway. The paw prints were then electronically archived through scanning. The distance between adjacent hind paw prints on the same side was measured as the stride length, while the width between the left and right hind paw prints was recorded as the step width.

### BMS scores

On the 7th, 14th, 21st, and 28th days post-surgery, the motor function of the mice was assessed using the Basso-Mouse Scale (BMS). The BMS has a total score of 9 points, with higher scores indicating better motor function recovery, 0 points indicating loss of hind limb motor function, and 9 points indicating fully normal motor function in both hind limbs.

### Statistical analysis

All experiments were performed at least three times. The results were presented as the mean ± standard deviation using SPSS v.20.0 software (SPSS, Inc., Chicago, USA), and graphs were generated with GraphPad Prism 8.0. Normality of data distribution was assessed using the Shapiro-Wilk normality test, and homogeneity of variance was evaluated using the Brown-Forsythe test for multiple groups. Student's t-test, one-way or two-way analysis of variance were used to compare data from different groups. Values of *P*<0.05 were considered statistically significant.

## Results

### Neuronal apoptosis increased accompanied with aggravated oxidative stress and inhibited mitophagy after SCI

**Figure 1:**
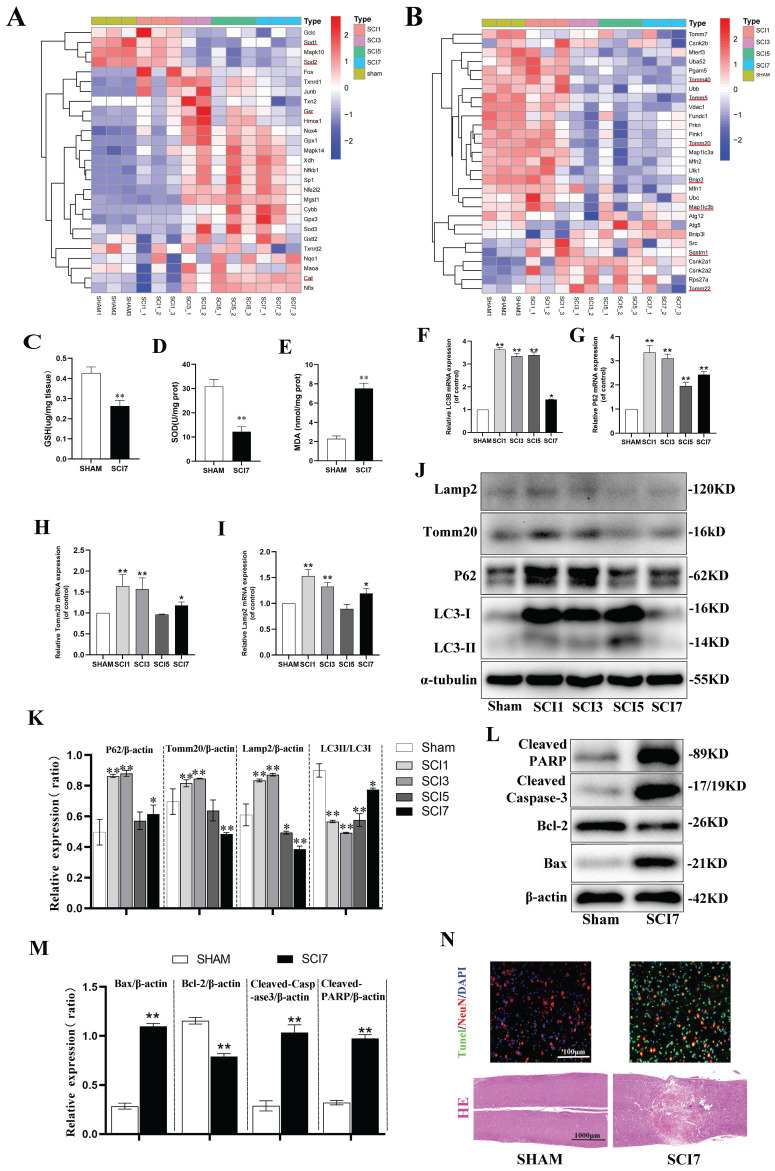
Neuronal apoptosis increased accompanied with aggravated oxidative stress and inhibited mitophagy after SCI. (**A, B**) Heatmap displays the changes of genes related to oxidative stress and mitophagy after 1, 3, 5, and 7 days following SCI. The typical genes of interest were marked with a red underline (One sample of SCI 3d failed to sequence, leaving us with only two samples in the SCI 3d group. n=3 animals for sham and SCI1,5,7d groups and n=2 animals for SCI3d group.). (**C-E**) GSH content, SOD activity, and MDA content were measured between sham and SCI7d groups (n=3 animals per group). (**F-I**) Relative mRNA changes of mitophagy-related genes LC3B, P62, Tomm20 and Lamp2 after 1, 3, 5, and 7 days following SCI (n=3 animals per group). (**J, K**) Representative western blot bands and the quantitative analysis of the corresponding grayscale values of mitophagy-related proteins LC3B, P62, Tomm20 and Lamp2 after 1, 3, 5, and 7 days following SCI (n=3 animals per group). (**L, M**) Representative western blot bands and the quantitative analysis of the corresponding grayscale values of apoptosis-related proteins BCL-2, BAX, Cleaved Caspase-3 and Cleaved PARP between sham and SCI7d groups (n=3 animals per group). (**N**) Representative images of HE and NeuN/Tunel staining between sham and SCI7d groups (n=3 animals per group). *P<0.05 and **P<0.01 versus sham group.

RNA-seq was performed to determine the transcriptomic landscape after SCI contusion injury. Hierarchical clustering of oxidative stress related genes showed SOD1 and SOD2, encoding for SOD, downregulated significantly from day 3 until day 7 after injury. Additionally, the genes encoding for Glutathione-Disulfide Reductase (GSR) and Catalase (CAT) exhibited significant upregulation from day 3 and 5 onwards (Fig. 1A). To further verify the sequence results, spinal cord samples were collected from mice in the sham and SCI 7d groups. Tissues were homogenized and protein supernatants were obtained for the assessment of SOD activity, GSH and MDA contents. It was found that compared to the sham group, the SCI 7d group exhibited a significant decrease in SOD activity, a notable decrease in GSH content, and a significant increase in MDA levels (Fig. 1C-E). These results indicate a significant enhancement of oxidative stress response following SCI.

In the hierarchical clustering analysis of mitophagy-related genes, it was observed that the expression of Sqstm1 (encoding the P62 protein) significantly increased from the first day after SCI, slightly decreased on the third day, and continued to decrease on the fifth and seventh days, reflecting a process of increasing expression in response to stress followed by gradual depletion. The autophagosome marker Map1lc3b (encoding the LC3B protein) showed a significant increase in expression from the first day after SCI, began to decrease on the third day, and exhibited fluctuations on the fifth and seventh days, but still showed a decreasing trend. The mitochondrial gene Tomm40 was significantly upregulated, Tomm20 remained unchanged or showed slight upregulation, and Tomm5 was slightly downregulated on the first day. Subsequently, sustained fluctuating decreases of these genes were observed on the third, fifth, and seventh days. The Tomm22 gene showed increased expression from the third day after SCI, with fluctuating decreases on the fifth and seventh days (Fig. 1B). RT-qPCR experiments revealed that relative P62 mRNA expression significantly increased on the first day, gradually decreased on the third and fifth days, and slightly increased again on the seventh day. The gene expression of LC3B remained high on the first, third, and fifth days, with a relatively unobvious decrease on the third and fifth days, but a significant decrease on the seventh day. The Tomm20 and Lamp2 genes both showed increased expression on the first day, moderate decreases on the third and fifth days, and a slight increase in expression on the seventh day (Fig. 1F-I). It can be observed that while the results of PCR and RNA sequencing are generally consistent in terms of overall trends, there are still differences in specific time points, which are likely due to differences between the two experimental systems and measurement methods. The final protein expressions were confirmed by western blot to further clarify the trend of mitophagy in the acute and subacute early stages after SCI (Fig. 1J, K). P62 protein showed significant accumulation on days 1 and 3 after SCI, which was gradually consumed and decreased on days 5 and 7. P62 protein is an autophagy protein with distinct substrate specificity and is degraded in the late stage of autophagy, thus showing a negative correlation with autophagic flux 49. Early accumulation of P62 suggests the intrinsic need for inducing autophagy, but the autophagic flux fails to be activated indeed. During autophagy, cytoplasmic LC3-I continuously converts to the membrane-bound LC3-II, promoting the continuous elongation of autophagosome membranes. Therefore, the ratio of LC3-II:LC3-I better reflects the autophagic flux 50. Although both PCR and western blot showed a significant increase in the total amount of LC3B on days 1, 3, and even 5 after SCI, the ratio of LC3-II:LC3-I decreased, indicating that cytoplasmic LC3-I did not fully convert to membrane-bound LC3-II during this period, thus failing to form effective autophagosomes. This also indicates that the intrinsic need for activation of autophagic flux was not fully realized. Mitochondrial membrane protein Tomm20 and lysosomal membrane protein Lamp2 also showed moderate accumulation in the early days of 1 and 3, and began to be slowly consumed on days 5 and 7. This suggests that the early dysfunctional mitochondria were not fully engulfed and degraded by lysosomes, resulting in the accumulation of corresponding proteins. Subsequently, a certain compensatory mitophagy was activated consuming certain amount of Tomm20 and Lamp2 (Fig. 1J, K). These results indicate that there is an intrinsic need to activate sufficient mitophagy in the early stages of SCI to clear dysfunctional mitochondria. However, in the acute and subacute early stages, mitophagy fails to be sufficiently activated and remains inhibited.

According to the clustering analysis of apoptosis-related genes, the anti-apoptotic gene Bcl2 was significantly downregulated on the first day, while the Bcl2l1 gene showed downregulation on the 5th and 7th days, and the downregulation of the Bcl2l2 gene began on the 3rd day and continued until the 7th day.

The pro-apoptotic genes BAX and Fas showed a slight increase on the first day, with a significant increase starting from the 3rd day until the 7th day. The apoptotic genes Caspase1, 2, 3, 4, 6, 7, 8, and 9 all showed significant upregulation from the 3rd day, continuing until the 5th and 7th days (Fig. S2). Based on the indications of the sequence results, samples from sham and SCI 7d group were collected for western blot and section staining. Compared to the sham group, the anti-apoptotic protein Bcl2 was significantly downregulated in the SCI7d group, while the pro-apoptotic protein BAX was significantly upregulated. The cleaved forms of Caspase3 (Cleaved Caspase-3) and PARP (Cleaved PARP) were both markedly upregulated (Fig. 1L, M). HE staining showed disordered and lighter staining in the injured tissues at day 7 post-SCI, with the appearance of vacuolization, but no large voids or defects. The Tunel staining of the tissues showed a significant increase in overall cell apoptosis (green Tunel-positive cells) and neuronal apoptosis (overlap of red NeuN and green Tunel, appearing yellow) (Fig. 1N). These results indicate a significant increase in neuronal apoptosis after SCI.

### Ligustilide attenuated oxidative stress-induced neuronal apoptosis

**Figure 2:**
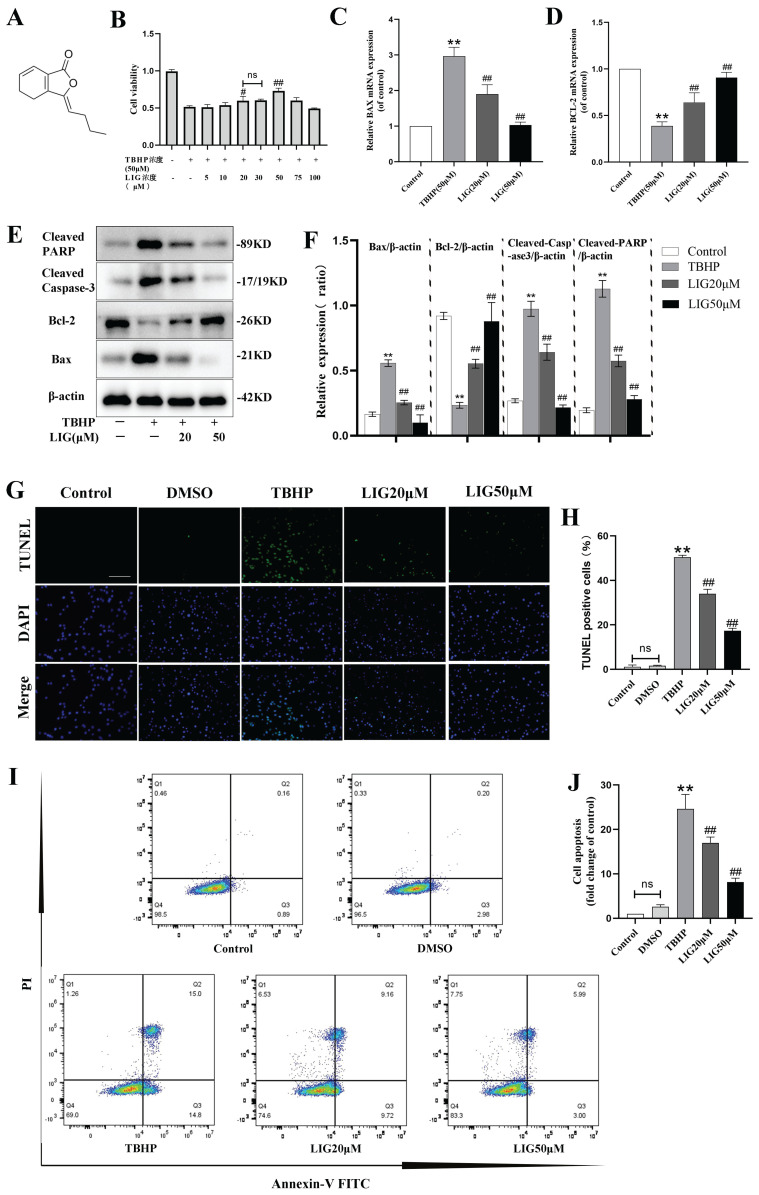
Ligustilide attenuated oxidative stress-induced neuronal apoptosis. (**A**) Chemical structure of ligustilide. (**B**) Effects of varying doses of ligustilide on the viability of PC12 cells treated with TBHP (50μM) (n=3). (**C, D**) Relative mRNA levels of BAX and BCL-2 in PC12 cells of each group detected by real-time PCR (n=3). (**E, F**) Representative western blot bands and quantitative analysis of apoptosis-related proteins in different groups (n=3). (**G, H**) Representative TUNEL images and corresponding quantifications of TUNEL positive cells per field in different groups, Scale bar :100μm, (n=5). (**I, J**) Cells were trypsinized and stained with Annexin V-FITC and PI. Apoptosis was analyzed by flow cytometry, and representative images of the cell population distribution were displayed. The sum of early(Q3) and late(Q2) apoptotic rate was identified as the total cell apoptosis which was used for quantification analysis (n=3). *P<0.05 and **P<0.01 versus control group, ^#^ p < 0.05 and ^##^ p < 0.01 versus TBHP group, ns: not significant.

Excessive accumulation of ROS is a critical pathophysiological hallmark of SCI 4. In order to imitate the oxidative damage observed in neurons during SCI, we established an *in vitro* model using PC12 cells treated with TBHP, a well-known inducer of ROS and oxidative stress. The cells exhibited a significant decrease in viability at 50 μM, which was identified as the optimal concentration for the cell model based on the demands of simulating peroxidation, as determined by the CCK-8 assay (Fig. S1A). LIG is one of the main active ingredients isolated from the traditional medicine of Umbelliferae family with a distinct chemical structure (Fig. 2A) and CCK-8 assay showed its cell protective effects at 20 μM and 50 μM with bare toxic effects (Fig. 2B, S1B). To confirm the apoptosis induced by TBHP and the corresponding anti-apoptotic effects of LIG, several experiments were conducted. Bax is a key apoptotic gene, while BCL-2 is anti-apoptotic. Caspase-3 plays a crucial role in apoptosis by cleaving several essential downstream substrates, including poly ADP-ribose polymerase (PARP). The activated forms, cleaved Caspase-3 and cleaved PARP, are strong indicators of apoptosis [51.52]. After treatment with 50μM TBHP, there was a significant increase in the mRNA expression of BAX and a noticeable decrease in BCL-2 expression, both of which were reversed to varying degrees by LIG, depending on the concentration of the dose (Fig. 2C, D). The corresponding effects were demonstrated in western blot bands, which also showed a remarkable increase in cleaved Caspase-3 and PARP after TBHP treatment, both of which were mitigated by different doses of LIG (Fig. 2E, F). TUNEL-positive cells, representing apoptotic cells, were markedly more frequent in the TBHP group than in the control and vehicle (DMSO) group. However, the addition of 20μM and 50μM LIG significantly decreased the positive rate (Fig. 2G, H). Additionally, using flow cytometry, we found that the total cell apoptosis rate was significantly higher in the TBHP group than in the control and DMSO group, and different doses of LIG reduced this (Fig. 2I, J). Collectively, these results suggest that LIG alleviated oxidative stress-induced neuronal apoptosis.

### Ligustilide reversed oxidative stress-induced mitophagy inhibition

**Figure 3:**
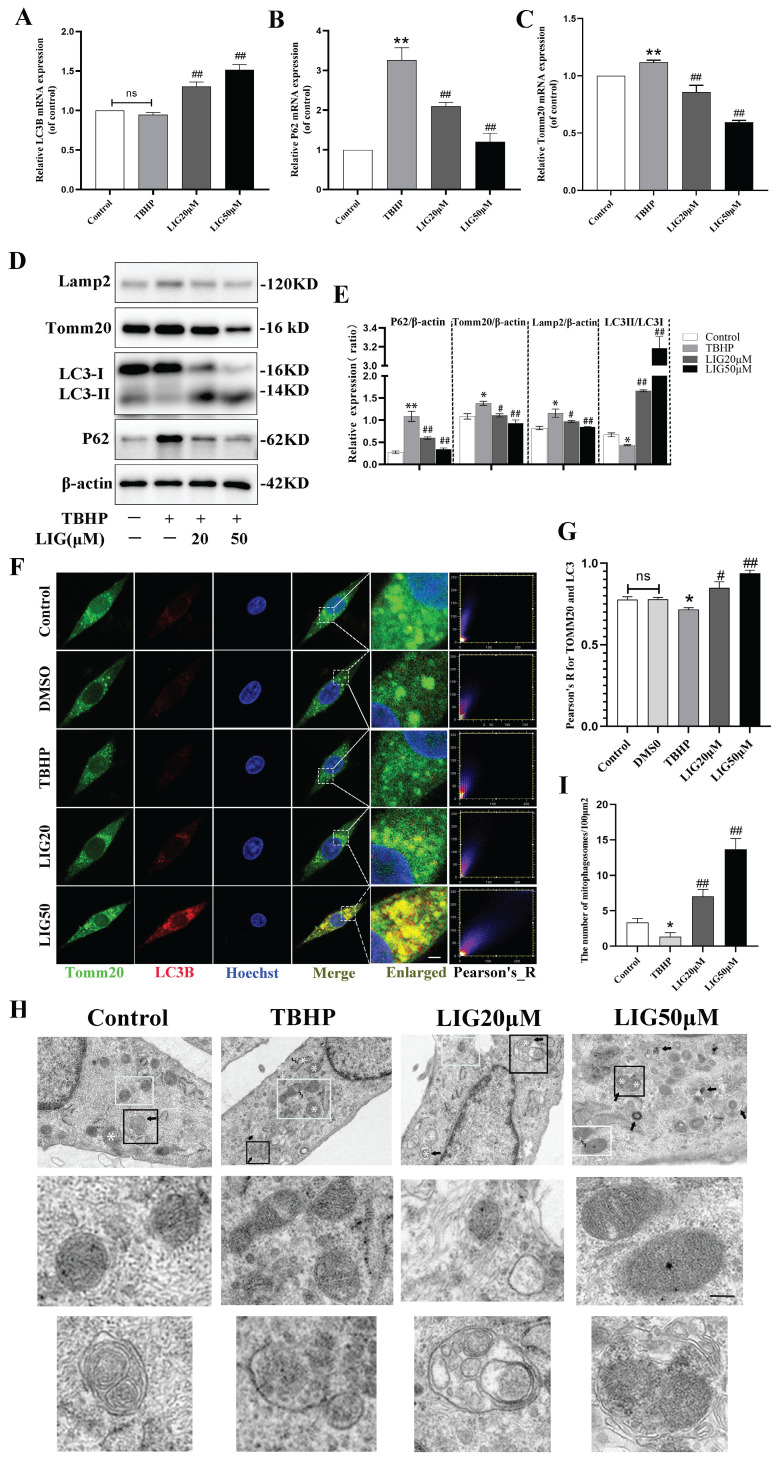
Ligustilide reversed oxidative stress-induced mitophagy inhibition. (**A-C**) Relative mRNA levels of LC3B, P62 and Tomm20 in PC12 cells with various treatment (n=3). (**D, E**) Representative western blot bands and quantitative analysis of mitophagy-related proteins (LC3, P62 Tomm20 and Lamp2) in different groups (n=3). (**F**) Representative fluorescence images with LC3B (red) and Tomm20 (green) double-staining and colocalization analysis using ImageJ colocalization finder for different cell groups, the Pearson's R for LC3B and Tomm20 was used for quantitative analysis (**G**), Scar bar: 5μm, n=5. (**H**) Transmission electron microscopy images of mitochondria and mitophagosomes in PC12 cells, and the corresponding quantitative analysis for the number of mitophagosomes (**I**). Asterisk: swollen mitochondria. Lightning mark: mitochondrial rupture. Arrow: double membrane autophagosome vacuoles encapsulating mitochondria. White frame: representative mitochondria magnified in the second row. Black frame: representative double membrane autophagosome vacuoles encapsulating mitochondria enlarged in the third row, Scar bar: 0.5 μm, n=3. *P<0.05 and **P<0.01 versus control group, ^#^ *P* < 0.05 and ^##^ *P* < 0.01 versus TBHP group, ns: not significant.

Suffering from a series of damage during SCI, there is an innate need in the spinal cord to activate various kinds of compensatory mechanisms and the mitophagy is a vital one 32-34. Due to the ROS overproduction causing oxidative damage to the biomembrane, the activation of mitophagy flux may be impeded. Our RNA sequencing results from animal samples have already indicated an increase in oxidative stress and a suppression of mitophagy flux. However, it remains unclear whether excessive ROS directly led to the inhibition of mitophagy. To address this and further clarify the role of LIG, several kinds of experiments were performed and mitophagy-related markers such as Lamp2, Tomm20, P62 and LC3 were detected *in vitro*. LC3B, an autophagosome marker, remained almost unchanged after TBHP treatment in gene expression (Fig. 3A), but the protein ratio of LC3Ⅱ:LC3Ⅰ decreased significantly in the TBHP group (Fig. 3D, E). After TBHP treatment, P62, a mitophagy adaptor oppositely changed with mitophagy flux, exhibited a significant increase, while Tomm20, a mitochondrial outer membrane protein, showed a moderate increase in both gene and protein expression (Fig. 3B-E).

Western blots also revealed that the lysosomal surface marker Lamp2 increased moderately in the TBHP group (Fig. 3D, E). Using immunofluorescence staining and confocal microscopy, we observed that TBHP attenuated the co-localization of LC3B and Tomm20 compared to the control and DMSO group (Fig. 3F, G). The mitophagy was further confirmed with TEM in each group. Compared with the healthy cells, cells treated with TBHP exhibited more swollen mitochondria manifested as the disappearance of mitochondrial cristae, and more mitochondrial ruptures, but rare double membrane autophagosomes encapsulating mitochondria (Fig. 3H, I). The above results demonstrate that mitophagy flux is inhibited in an excessive ROS microenvironment. After the addition of 20μM and 50μM LIG, the mRNA expression of LC3B increased, at the same time P62 and Tomm20 decreased gradually (Fig. 3A-C). Simultaneously, the protein expression of LC3Ⅱ:LC3Ⅰ markedly increased, accompanied by a gradual decrease in Tomm20, Lamp2, and P62 (Fig. 3D, E). The co-localization of LC3B and Tomm20 was gradually enhanced as the LIG dose increased (Fig. 3F, G). TEM images further confirmed that there were more mitophagosomes, and those mitochondria that had not been engulfed were healthier in the LIG-treated groups, especially in the LIG50μM group, than in the TBHP group (Fig. 3H, I). Taken together, these results demonstrate that LIG reversed and further enhanced the mitophagy flux inhibited by excessive oxidative stress.

### Ligustilide alleviated oxidative stress-induced mitochondrial dysfunction

**Figure 4:**
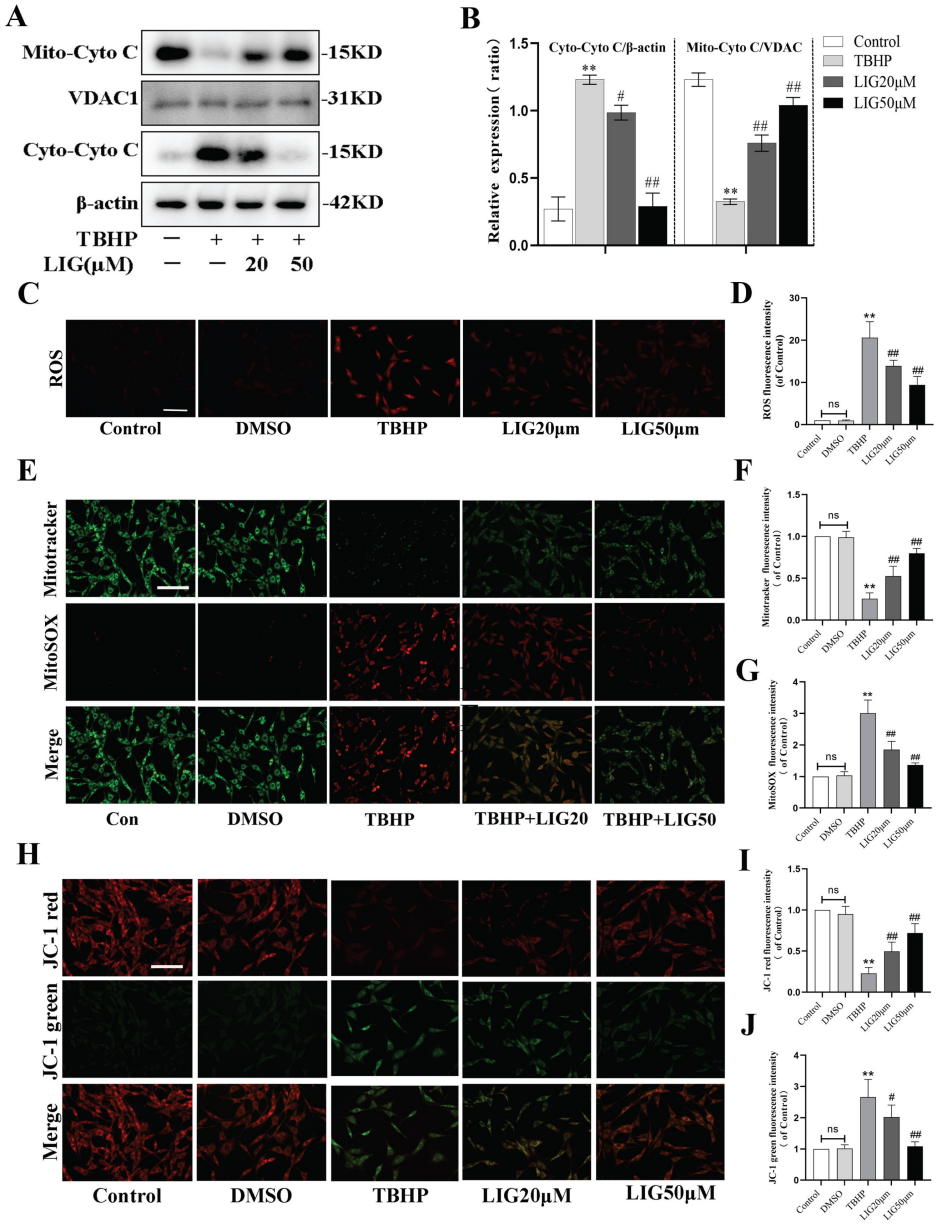
Ligustilide alleviated oxidative stress-induced mitochondrial dysfunction. (**A, B**) Representative western blot bands and quantification analysis showing the mitochondrial and cytoplasmic Cytochrome C changes in different cell groups (n=3). (**C, D**) Representative micrographs of DHE (red) staining whose intensity represents the levels of ROS in different cell groups and the quantitative analysis of ROS fluorescence intensity. Scar bar: 200μm, n=5. (**E-G**) Representative fluorescence images with Mito-SOX (red) and Mito-Tracker (green) double-staining and the quantitative analysis. Scar bar:50μm, n=5. (**H-J**) The MMP was detected through JC-1 staining and the corresponding quantitative analysis was conducted for JC-1 red and green respectively. Scar bar:50μm, n=5. *P<0.05 and **P<0.01 versus control group, ^#^ *P* < 0.05 and ^##^ *P* < 0.01 versus TBHP group.

Excessive ROS production leads to defective mitophagy. To further investigate whether it causes mitochondrial dysfunction and the effect of LIG, 50μM TBHP was administrated to induce excessive ROS and the total ROS production was detected by dihydroethidium (DHE) staining. According to the fluorescence images, the fluorescence intensity parallel with ROS levels was remarkably enhanced by TBHP compared with the control and DMSO group, while the addition of LIG obviously attenuated the ROS intensity especially the concentration of 50μM (Fig. 4C, D). Mitochondria are known to be the main source of endogenous ROS due to their respiratory chain 14. To detect mitochondria-specific ROS, cells were treated with both MitoSOX Red and MitoTracker Green. The oxidative damage induced by TBHP also significantly increased the mitochondria-specific ROS, which was gradually reduced by varying doses of LIG. The intensity of MitoTracker Green changed in the opposite direction to MitoSOX (Fig. 4E-G). Cytochrome C (Cyto C), a protein of mitochondrial respiratory chain, will be released to cytoplasm when mitochondria are damaged 53. Western blots exhibited an obvious translocation of Cyto C from mitochondria to cytoplasm after TBHP administration, which was dramatically reversed by LIG (Fig. 4A, B). MMP is another critical indicator of mitochondrial function, and decreased MMP is closely associated with mitochondrial dysfunction 54. JC-1 staining was performed to detect the MMP changes in each group. The images showed remarkable conversion from red fluorescence to green in the TBHP group compared with control and DMSO group, and the administration of LIG gradually reversed the conversion especially in the LIG50μM group (Fig. 4H-J). Collectively, these results suggest that excessive ROS production induced by TBHP caused mitochondrial dysfunction which was alleviated by LIG.

### Ligustilide attenuated oxidative stress-induced neuronal apoptosis partially through enhancing mitophagy

**Figure 5:**
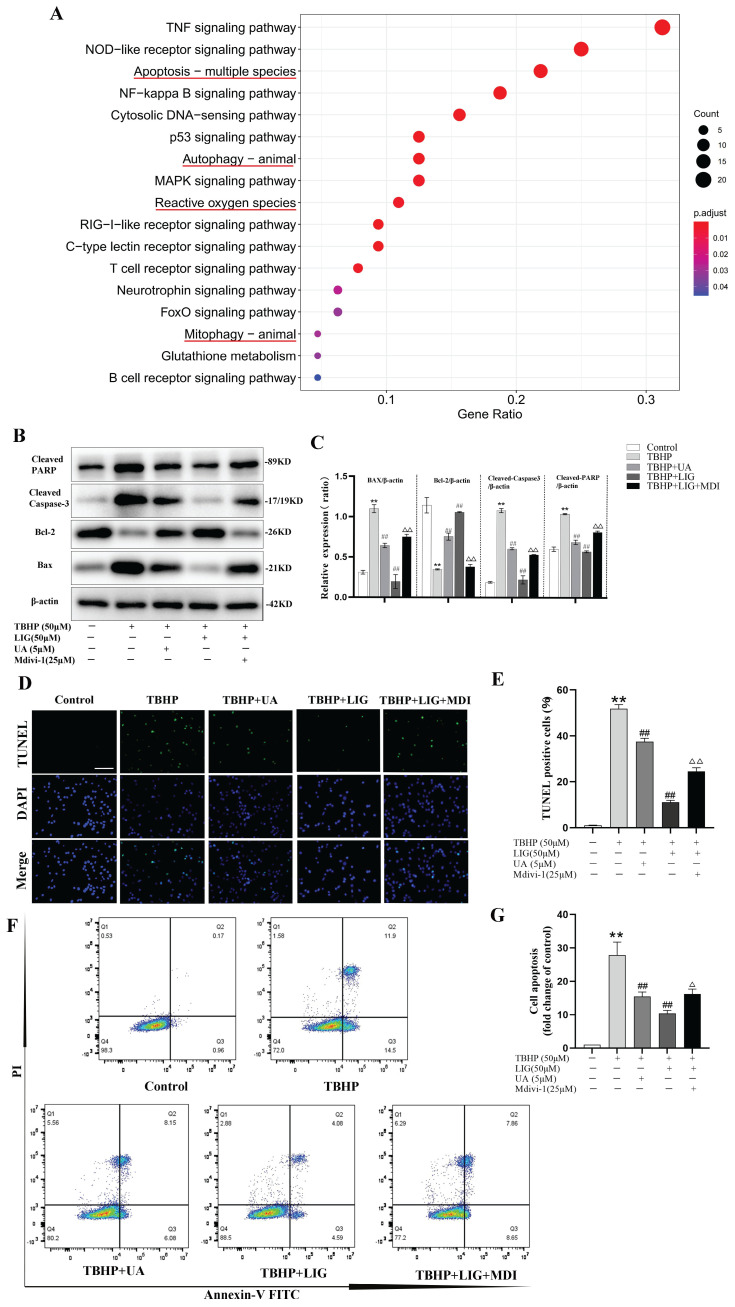
Ligustilide attenuated oxidative stress-induced neuronal apoptosis partially through enhancing mitophagy. (**A**) Kyoto Encyclopedia of Genes and Genomes (KEGG) enrichment analysis of DEGs between samples of sham and SCI5d (n=3 animals per group). The enriched pathways of interest were highlighted with a red underline. (**B, C**) Western blot and densitometric analysis of apoptosis-related proteins in PC12 cells treat with TBHP(50μM), ligustilide(50μM), UA(urolithin A, a mitophagy agonist, 5μM), and Mdivi-1(a selective mitophagy inhibitor, 25μM) (n=3). (**D, E**) Representative TUNEL images and corresponding quantifications of TUNEL positive cells in different groups. Scale bar :50μm, n=5. (**F, G**) Cells of each group were trypsinized and stained with Annexin V-FITC and PI. Apoptosis was analyzed by flow cytometry, and representative images of the cell population distribution were displayed with their quantitative analysis (n=3). *P<0.05 and **P<0.01 versus the control group, ^#^ *P* < 0.05 and ^##^ *P* < 0.01 versus with the TBHP group, ^△^*P* < 0.05 and ^△△^*P* < 0.01 versus the TBHP+LIG group.

Apoptosis, autophagy, mitophagy, and ROS-related gene sets are all significantly enriched, as indicated by the KEGG and GO enrichment analysis of DEGs between the sham and SCI5d samples (Fig. 5A, S3). Given that mitophagy is a vital mechanism functioning to maintain mitochondrial quality control and homeostasis 13, we speculate that enhancing mitophagy attenuates neuronal apoptosis, which may be the potential mechanism of LIG against apoptosis. To clarify this, Mdivi-1, a well-known selective mitophagy inhibitor, and UA, a proven beneficial mitophagy agonist 48, were administered *in vitro*. Compared to UA, the addition of LIG based on TBHP exhibited similar but much stronger anti-apoptotic effects as evidenced by decreased BAX, cleaved Caspase-3, cleaved PARP protein expression and increased BCL-2 protein expression (Fig. 5B, C), as well as reduced TUNEL-positive cells per field (Fig. 5D, E) and apoptotic cell distribution in the Q2 and Q3 region (Fig. 5F, G). Based on the co-administration of TBHP and LIG, the addition of Mdivi-1 partially abrogated the anti-apoptotic effects of LIG, manifested as apoptotic markers increased and anti-apoptotic markers decreased again as shown in Fig. 5B, C. TUNEL and Flow Cytometry results were consistent with western blots in the TBHP+LIG+MDI group compared to the TBHP+LIG group (Fig. 5D-G). Taken together, the activation of mitophagy attenuated oxidative stress-induced neuronal apoptosis, and the anti-apoptotic effects of LIG were partially through enhancing mitophagy.

### LIG enhanced mitophagy through BNIP3-LC3 interaction

**Figure 6:**
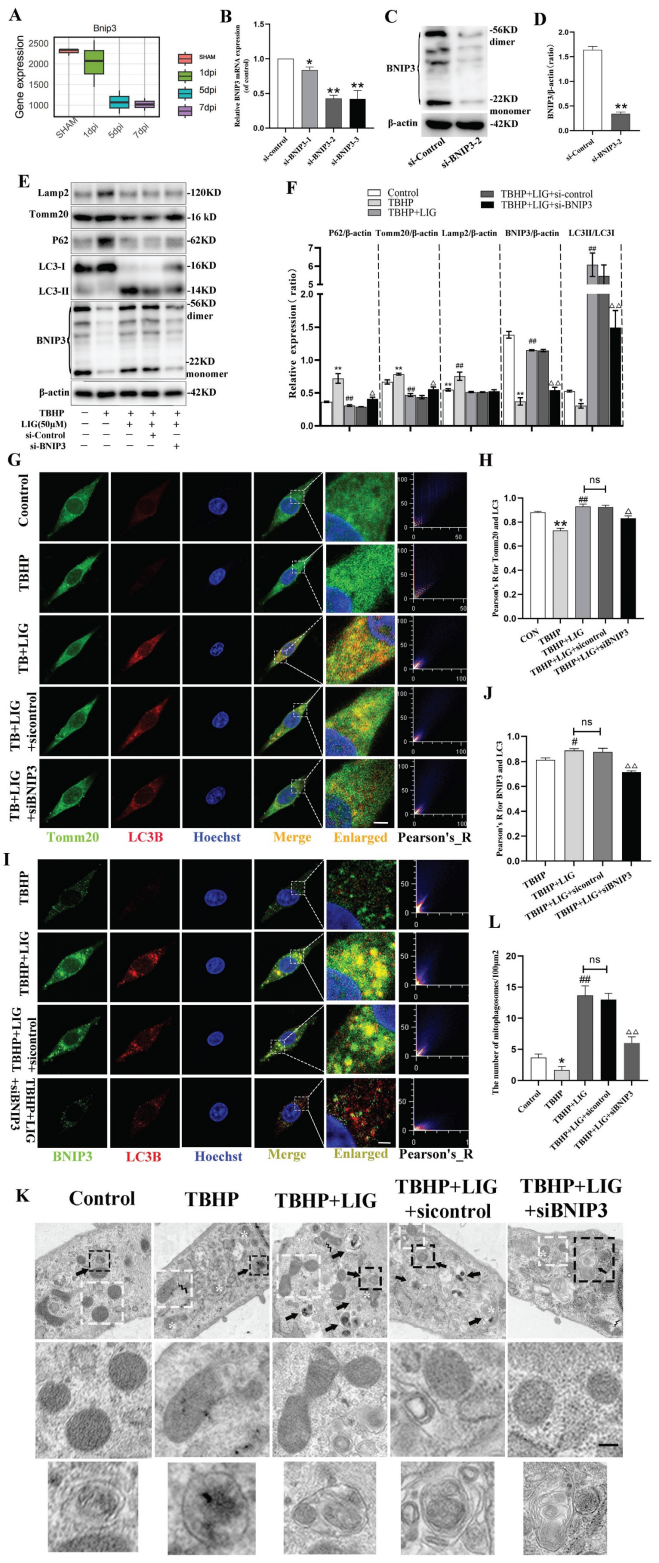
Ligustilide enhanced mitophagy through BNIP3-LC3 interaction. (**A**) The boxplot illustrates the expression changes of BNIP3 at 1, 5, and 7 days post-SCI (n=3 animals per group). (**B-D**) Three siRNA sequences for silence BNIP3 were transfected into PC12 cells and the most effective sequence was selected through RT-qPCR and confirmed by Western blot (n=3). (**E, F**) Representative western blot bands and quantitative analysis of LC3, P62, Tomm20, Lamp2 and BNIP3 in Control, TBHP, TBHP+LIG, TBHP+LIG+siControl and TBHP+LIG+siBNIP3 groups (n=3). (**G**) Representative fluorescence images with LC3B (red) and Tomm20 (green) double-staining and colocalization analysis using Image J colocalization finder for different cell groups, the Pearson's R for LC3B and Tomm20 was used for quantitative analysis (**H**). Scar bar: 5μm, n=5. (**I**) Representative fluorescence images with LC3B (red) and BNIP3 (green) double-staining and colocalization analysis using ImageJ colocalization finder for different cell groups, the Pearson's R for LC3B and BNIP3 was used for quantitative analysis (**J**). Scar bar: 5μm, n=5. (**K**) Transmission electron microscopy images of mitochondria and mitophagosomes in different cell groups, and the corresponding quantitative analysis for the number of mitophagosomes (**L**). Asterisk: swollen mitochondria. Lightning mark: mitochondrial rupture. Arrow: double membrane autophagosome vacuoles encapsulating mitochondria. White frame: representative mitochondria magnified in the second row. Black frame: representative double membrane autophagosome vacuoles encapsulating mitochondria enlarged in the third row (Scar bar: 0.5 μm, n=3). *P<0.05 and **P<0.01 versus the control group, ^#^ *P* < 0.05 and ^##^ *P* < 0.01 versus with the TBHP group, ^△^*P* < 0.05 and ^△△^*P* < 0.01 versus the TBHP+LIG group, ns: not significant.

In general, the regulation of mitophagy can be divided into parkin RBR E3 ubiquitin protein ligase (PRKN)-dependent and PRKN-independent pathways 32. To further explore the underlying mechanism of mitophagy involved in SCI, the RNA sequence data was carefully scanned. Interestingly, hierarchical clustering of both mitophagy- and apoptosis-related genes revealed that BNIP3 began to be downregulated on the first day after SCI, with the downregulation intensifying on the third day and persisting through the fifth and seventh days (Fig. 1B, S2). Then, separate statistical analyses were conducted on the BNIP3 gene in each sequencing sample, confirming consistent alterations (Fig. 6A). These results indicated that BNIP3 might be the potential target of mitophagy involved in SCI. To confirm this, siRNA sequences were constructed to silence BNIP3. RT-qPCR was performed to select the best sequence (Fig. 6B) and western blot further confirmed the effectiveness (Fig. 6C, D). The administration of TBHP induced significant decrease of BNIP3, accompanied with the increase of P62 and Tomm20, but unsignificant decrease of LC3B in mRNA expression (Fig. S4A-D). Western blot confirmed the decrease of BNIP3 and the accumulation of P62, Tomm20 and Lamp2, but a significant decrease of LC3Ⅱ:LC3Ⅰ in protein expression (Fig. 6E, F). The addition of 50μM LIG reversed the TBHP-induced inhibition of mitophagy, as evidenced by the increased expression of BNIP3, LC3B, LC3Ⅱ:LC3Ⅰ, and decreased levels of P62, Tomm20, and Lamp2 (Fig. 6E, F; S4A-D). Based on the co-administration of TBHP and LIG, transfection of a negative control sequence didn't trigger any changes in both mRNA and protein expressions. But the addition of siBNIP3 not only led to a decrease in BNIP3, but also a reduction in LC3Ⅱ:LC3Ⅰ and an accumulation of P62, Tomm20, and Lamp2 compared to the TBHP+LIG group (Fig. 6E, F; S4A-D). The LIG-enhanced co-localization of LC3B and Tomm20 was attenuated by the additional siBNIP3 transfection (Fig. 6G, H). To further determine whether LIG-enhanced mitophagy was through BNIP3-LC3 interaction, the co-localization of BNIP3 and LC3B was detected by immunofluorescence staining and observed by confocal microscopy. Based on TBHP administration, the co-localization of BNIP3 and LC3B was enhanced by the addition of LIG, which was attenuated by further transfection of siBNIP3 (Fig. 6I, J). According to TEM analysis, cells in the TBHP group exhibited more swollen mitochondria, characterized by the disappearance of mitochondrial cristae and increased mitochondrial ruptures, but rare mitophagosomes were observed. With the addition of LIG, mitophagosomes increased, and mitochondria that were not engulfed appeared healthier. However, these therapeutic effects were partially abrogated by transfection of siBNIP3, as evidenced by reduced mitophagosome numbers and vague mitochondrial structures (Fig. 6K, L). Taken together, these results demonstrate that BNIP3 downregulation participates in the SCI-induced mitophagy inhibition and that LIG enhances mitophagy through BNIP3-LC3 interaction.

### Ligustilide alleviated oxidative stress, enhanced mitophagy, and attenuated neuronal apoptosis* in vivo*

**Figure 7:**
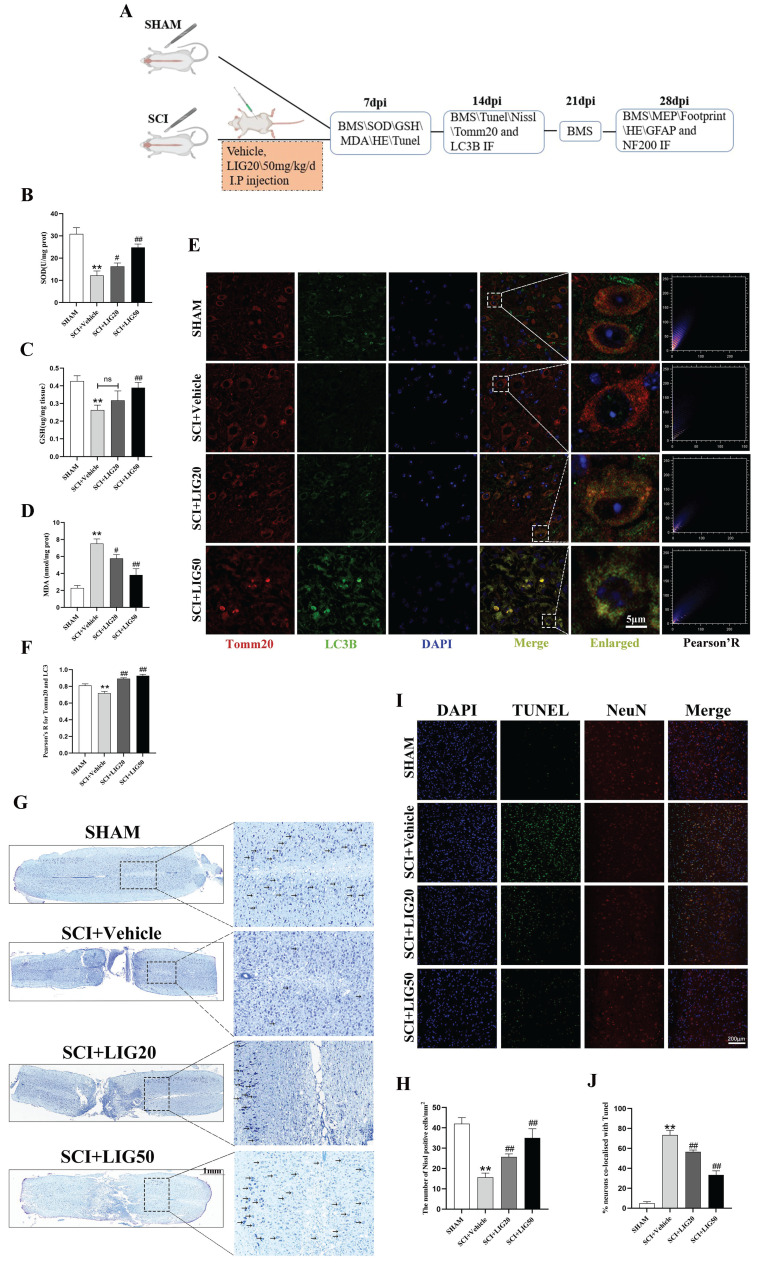
Ligustilide alleviated oxidative stress, enhanced mitophagy, and attenuated neuronal apoptosis *in vivo*. (**A**) The flowchart for grouping, handling, and specimen testing of mice. (**B-D**) Spinal cord SOD activity, GSH content, and MDA content in different groups (n=3 animals per group). (**E, F**) Representative immunofluorescence images of Tomm20 and LC3 in each group and colocalization analysis using ImageJ colocalization finder. Scale bar: 5μm, n=6 animals per group. (**G**, **H**) Representative images of Nissl staining in the spinal cord of each group and quantitative analysis of the number of Nissl bodies per square millimeter. Scale bar: 1mm, n=6 animals per group. (I**, J**) Representative images of spinal cord Tunel/NeuN/DAPI staining in each group and quantitative analysis of neuronal apoptosis. Scale bar: 200μm, n=6 animals per group. *P<0.05 and **P<0.01 versus SHAM group, ^#^ *P* < 0.05 and ^##^ *P* < 0.01 versus SCI+Vehicle group, ns: not significant.

The animal experiments conducted at different time points were exhibited as the flowchart (Fig. 7A). SCI+Vehicle group exhibited a significant decrease in GSH content and SOD activity compared to the SHAM group, along with a marked increase in MDA content. However, SCI+LIG20 and SCI+LIG50 groups showed a gradual recovery in GSH content and SOD activity compared to the SCI+Vehicle group, accompanied by a gradual decrease in MDA content. Among them, the SCI+LIG50 group exhibited the most significant changes (Fig. 7B-D). Baseline levels of mitophagy were maintained in the sham group, as evidenced by a certain level of fluorescence intensity of LC3B and its co-localization with Tomm20, whereas these levels were decreased in the SCI+Vehicle group and gradually increased with the treatment of different doses of LIG (Fig. 7E, F). Nissl bodies are basophilic granules found in the bodies or dendrites of neurons, primarily composed of rough endoplasmic reticulum and free ribosomes. When neurons are damaged or over-fatigued, Nissl bodies decrease or disappear, but during the recovery process, Nissl bodies reappear and increase 55. Our Nissl staining results showed that tissue in the sham group had clear structure, with large and numerous Nissl bodies in the cytoplasm, distributed densely and evenly, and stained deep blue or purple. After SCI, tissue at the injury site was disrupted, and the number of Nissl bodies around the injury area also decreased apparently. Treatment with different doses of LIG promoted tissue repair in the injury area to varying degrees and resulted in a recovery in the number of Nissl bodies around the lesion site (Fig. 7G, H). According to the Tunel/NeuN/DAPI staining and the corresponding quantitative analysis for neurons co-localized with Tunel (Fig.7I, J), SCI increased neuronal apoptosis remarkably, which was gradually attenuated by the treatment with different doses of LIG. Collectively, these results demonstrate that LIG alleviated oxidative stress, enhanced mitophagy, ameliorated neuronal function and attenuated neuronal apoptosis *in vivo*.

### Ligustilide promoted tissue repair and functional recovery after SCI

**Figure 8:**
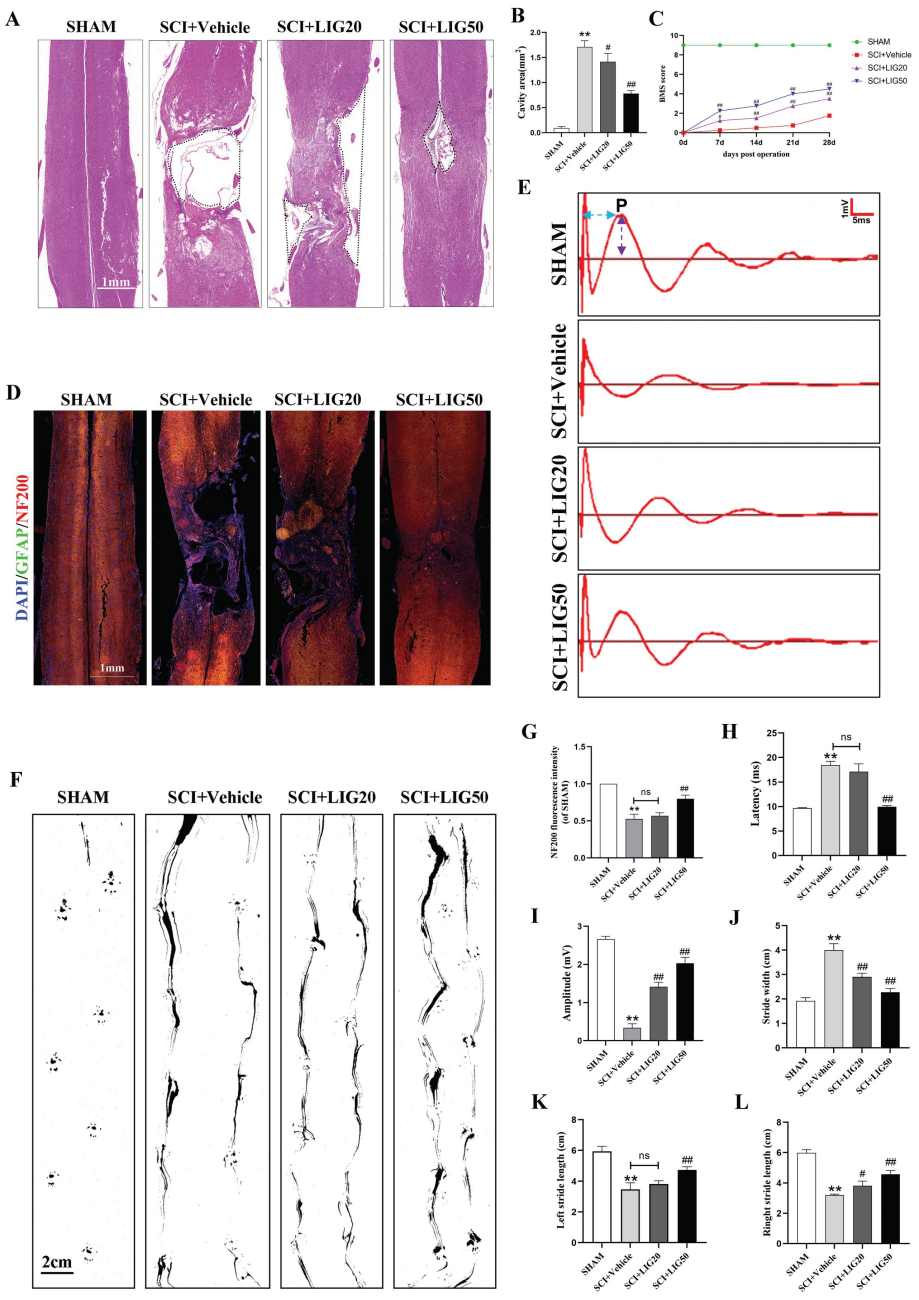
Ligustilide promoted tissue repair and functional recovery after SCI. (**A, B**) Representative images of HE staining in the spinal cord of each group and quantitative analysis of the corresponding cavity area. Scale bar: 1mm, n=6 animals per group. (**C**) The BMS scores at different time points for each group (n=12 animals per group). (**D, G**) Representative immunofluorescent images of GFAP/NF200/DAPI for each group and the corresponding quantitative analysis for the fluorescence intensity of NF200. Scale bar: 1mm, n=6 animals per group. (**E, H, I**) Representative waveforms of motor evoked potentials (MEP) from each group and quantitative analysis of the corresponding P-wave latency and amplitude (n=6 animals per group). (**F, J-L**) Representative hind paw footprints from each group and corresponding quantitative analysis of paw width and stride length of both sides. Scale bar: 2cm, n=12 animals per group. *P<0.05 and **P<0.01 versus SHAM group, ^#^ *P* < 0.05 and ^##^ *P* < 0.01 versus SCI+Vehicle group, ns: not significant.

According to the HE staining, spinal cord showed obvious cavities and defects in the injury site, with the surrounding area exhibiting loose staining, scattered vacuolar degeneration, and disorganized tissue structure in the SCI+vehicle group. After treatment with different doses of LIG, the area of cavities and defects was gradually shrinking, with regenerating spinal cord tissue crossing the injury site. The arrangement of repaired tissue was becoming increasingly orderly, with the SCI+LIG50 group showing the most significant improvement (Fig. 8A, B). Immunofluorescent staining with DAPI/GFAP/NF200 was further performed in the tissue sections to determine axon regeneration. An increasing amount of red fluorescence (NF200) was observed in the injury site after treatment with different doses of LIG, indicating varying degrees of axon regeneration, accompanied by increasingly dense and regular tissue repair (Fig. 8D, G). It remained unclear whether tissue repair led to functional recovery, and various experimental tests were performed to address this issue. Excitation of the motor cortex by electrical or magnetic stimulation leads to depolarization of spinal anterior horn cells or peripheral motor fibers. The resulting electrical potential recorded on the surface of muscles or nerves is referred to as MEP. MEP is an objective and reliable assessment indicator for motor function recovery after SCI, particularly the P-wave, which has a short latency period and is minimally affected by anesthetic drugs 56. Different groups of animals were subjected to MEP analysis. The results showed the latency of the P-wave was significantly prolonged and the amplitude was markedly reduced in the SCI+Vehicle group compared to the SHAM group. Treatment with LIG at a dose of 20mg/kg/d slightly shortened the latency of P-wave with no statistical difference, but the high dose of 50mg/kg/d significantly did with obvious statistical difference compared to the SCI+Vehicle group. The P-wave amplitude was significantly increased in both the LIG-treated groups compared to the vehicle treatment group, with the high dose resulting in an even greater increase (Fig.8E, H, I). Footprint analysis was performed to further confirm the motor function recovery of hind foot. As indicated in Fig. 8F, hind feet of mice in the SCI+Vehicle group showed obvious dragging during walking, with continuous ink marks visible between adjacent footprints, indicating that both hind feet were unable to completely leave the paper during walking, and the stride length was significantly shortened compared to the SHAM group, while the stride width increased. After treatment with 20mg/kg/d LIG, although the gait still showed dragging, the stride width narrowed (with statistical significance), and the stride length increased (The right side shows statistical significance, while the left side does not). In the 50mg/kg/d group, walking dragging improved. The stride width decreased and the stride length of both sides increased with all the differences being statistically significant compared to the SCI+Vehicle group (Fig. 8F, J-L). Finally, animals at 7,14, 21, 28 days post injury, were all subjected to BMS scale. After continuous administration for 7 days, both the SCI+LIG20 and SCI+LIG50 groups began to exhibit higher BMS scores than the SCI+Vehicle group. Over the following 2, 3, and 4 weeks, the BMS scores of the LIG treatment groups were all significantly higher than those of the Vehicle treatment group, with the SCI+LIG50 group showing the highest scores. By the end of the 4-week, the average BMS score of the SCI+LIG50 group was above 4, while that of the SCI+LIG20 group was above 3, and the average score of the SCI+Vehicle group was still below 2. These differences were all statistically significant (Fig. 8C). The aforementioned results demonstrate that LIG not only promoted tissue repair but also facilitated motor function recovery after SCI.

## Discussions

SCI triggers a cascade of progressive pathophysiologic changes. SOD activity and GSH content decreased obviously, whereas MDA content increased markedly in the acute and subacute early phase of SCI (Fig. 1C-E) indicating a decrease in the ability to clear free radicals and an increase in lipid peroxidation (LP) products. Continuous progression of LP leads to a decrease in MMP, insufficient ATP generation, sustained opening of the mitochondrial permeability transition pore, disruption of membrane integrity, and initiation of cellular apoptosis 57-59.

Research has reported that autophagic markers (LC3 and P62) increased and mitophagosomes appeared in the early stages of secondary injury 26-28, 60, 61, indicating that there is an intrinsic need to activate sufficient mitophagy to engulf dysfunctional mitochondria, complete mitochondrial quality control, and ultimately promote cell survival after SCI. Unfortunately, neither in animal models nor in cell experiments was any evidence found of fully activated mitophagy in the present study. Although total LC3 was remarkably upregulated at the acute or subacute early phase of SCI (Fig. 1B, F), the ratio of LC3Ⅱ:LC3Ⅰ decreased, and P62 increased, with Tomm20 and lamp2 moderately elevated (Fig. 1B, G-K; Fig. 3B-E), indicating that mitophagy flux was indeed inhibited. However, previous studies have reported an increase in levels of autophagic markers (LC3 and P62), and autophagosomes containing damaged mitochondria were found in injured neurons in the early stages of secondary injury. They believed mitophagy was excessively activated following SCI 61-63. But in our opinions, these research findings were largely consistent with our results. However, in interpreting mitophagy flux, it is not only important to observe the aggregation of LC3, P62, and the appearance of mitophagosomes, but also to determine whether the mitophagosomes ultimately successfully fuse with lysosomes and are degraded, thereby completing the entire process of mitophagy. Any blockade at any stage will preclude the designation of mitophagy activation. Total LC3 increased accompanied with LC3Ⅱ:LC3Ⅰ decreased implied that cytoplasmic LC3-I was unable to fully extend and convert into the membrane-bound LC3-II, thus failing to form functional autophagosomes. The aggregation of P62 indicated that the autophagosome did not fully fuse with the lysosome and was not degraded ultimately. The mild elevation of Tomm20 and Lamp2 might represent the aggregation of dysfunctional mitochondria and lysosomes that failed to be consumed through mitophagy. Taken together, mitophagy flux is actually inhibited at the acute or subacute early phase of SCI or after treatment with TBHP. We speculate that there may be two reasons: first, excessive ROS and LP after SCI damage the biological membranes, including the autophagosome and lysosome membranes, preventing their successful fusion and degradation; second, oxidative stress impairs mitochondrial function and hinders ATP production, leading to insufficient cellular metabolic energy supply. Additionally, we observed a decrease in P62, an increase in LC3-II:LC3-I, as well as a reduction in Lamp2 and Tomm20 on days 5 and 7 post-SCI (Fig. 1G-K) indicating that certain compensatory mitophagy was activated over time degrading a certain amount of Tomm20 and Lamp2.

Neurons are highly specialized cells, which are a basic structural and functional unit of the nervous system, and have roles in sensing stimulation and conducting excitation 64. Neuronal apoptosis plays a crucial role in secondary injury 5,65, results in neurological dysfunction and is associated with poor prognosis 66,67. Reducing neuronal apoptosis can promote neurorehabilitation, neuroplasticity, and axonal regeneration, and has become a strategy for treatment of SCI 68. Various strategies such as inhibiting neuroinflammation, alleviating endoplasmic reticulum stress and enhancing autophagy were performed to attenuate neuronal apoptosis 69-77. In addition, increasing ROS and mitochondrial dysfunction were reported leading to neuronal apoptosis and directly promoting SCI pathology progression 78-80. In the present study, our results demonstrated excessive ROS induced obvious mitochondrial dysfunction and remarkable neuronal apoptosis. LIG, one of the main active components isolated from traditional Chinese medicinal herbs such as Ligusticum chuanxiong and Angelica sinensis, is structurally similar to DL-3-n-butylphthalide (NBP), the first innovative drug in China with independent intellectual property rights and widely used for the treatment of ischemic stroke. Studies have shown that LIG has neuroprotective effects and the ability to penetrate the blood-brain barrier 39, 40. It exhibited anti-inflammatory 41,42, antioxidant, and anti-apoptotic effects 43,44 in central nervous system injuries and neurodegenerative diseases. Consistent with previous reports, our results also indicated that LIG significantly reduced excessive ROS production, and further attenuated oxidative stress-mediated mitochondrial dysfunction and neuronal apoptosis.

Based on our above findings, it is crucial to explore the underlying mechanism of oxidative stress-induced neuronal apoptosis, which may supply potential therapeutic targets for SCI. Mitophagy is a self-protective mechanism and selectively clears damaged or dysfunctional mitochondria through autophagic machinery. Therefore, mitochondrial dysfunction induced by oxidative stress has the potential possibility to activate mitophagy but failed to do this ultimately according to our above results. So, enhancing mitophagy to reduce neuronal apoptosis seems a reasonable and attractive strategy. Previous studies have shown that autophagy is inhibited after SCI thereby inducing cell death of injured neurons and the deterioration of SCI. Autophagy activators could significantly improve this situation and prevent neuron death 81-83. Appropriate autophagy activation could reduce inflammation and inhibit apoptosis 31,84,85, while autophagy inhibition rendered cells unable to cope with stressful situations and led to apoptosis 22,86-88. In the present study, UA, the mitophagy inducer, was employed to reverse the mitophagy inhibited by oxidative stress, and reduce neuronal apoptosis. As early as 2016, UA was demonstrated to induce mitophagy, increase the lifespan of C. elegans, and enhance muscle function in rodents 48. It was later confirmed to improve obesity-induced metabolic cardiomyopathy in mice by activating mitophagy 47. Thus, unlike other inducers such as Carbonyl Cyanide 3-ChloroPhenylhydrazone (CCCP) that activate mitophagy by promoting mitochondrial oxidative stress, fission, increasing mitochondrial permeability, and altering MMP 89-92, UA exhibits more beneficial effects. Our results demonstrated that the addition of UA, similar to the addition of LIG, successfully reversed TBHP-induced neuronal apoptosis, providing preliminary evidence that oxidative stress-mediated mitophagy inhibition following SCI directly leads to neuronal apoptosis, and LIG reverses this inhibition to alleviate apoptosis. Mdivi-1 is a selective inhibitor of dynamin-related protein 1 (Drp1) and can effectively inhibit mitophagy. Building on the reversal by LIG, the further addition of Mdivi-1 partially counteracted the anti-apoptotic effect of LIG, further confirming that LIG attenuates oxidative stress-mediated neuronal apoptosis by enhancing mitophagy. However, the anti-apoptotic effect of LIG was not completely counteracted by Mdivi-1, suggesting that there may be other mechanisms through which LIG mitigates apoptosis.

Constrained by multiple stressors, autophagy was reported exhibiting inconsistent and even contradictory manifestations at different times and in different injury models after SCI 24. Different injury models triggered varying degrees of autophagy 25. Strategies to enhance or inhibit mitophagy have been reported to target the repair of SCI, reflecting the inconsistent roles played by mitophagy in different spatiotemporal conditions of SCI. Schwann cell-derived exosomes, rapamycin, maltol, apelin-36, and zinc have all been reported to possess the ability to enhance mitophagy and alleviate SCI 9,93-96. Conversely, strategies to inhibit mitophagy to improve SCI have also been reported. Rosiglitazone and salidroside were reported to improve SCI by inhibiting mitophagy 97, 98. We believe that whether to enhance or inhibit mitophagy to target SCI mainly depends on different injury severities and times. In our mouse spinal cord contusion model, we found that mitophagy inhibition occurred in the acute and subacute early stages of injury, and signs of compensatory enhancement of mitophagy appeared from the 5th day of injury. Our drug intervention in mice was also targeted at 1 to 7 days after injury, achieving good tissue and function recovery. Therefore, only by thoroughly studying the evolving trends of mitophagy at different injury severities and stages, and taking targeted interventions, can better repair effects be achieved.

The potential mechanisms underlying mitophagy inhibition during the acute and subacute phases of SCI, as well as under excessive ROS stimulation *in vitro*, and the pathway through which LIG enhances mitophagy, are worthy of further investigation. Both BNIP3 and BCL2/adenovirus E1B interacting protein 3-like (BNIP3L) contain LC3-interacting regions (LIRs) and can directly bind to LC3-GABARAP, inducing mitophagy without the need for ubiquitination process 99. BNIP3/BNIP3L is located on the outer membrane of mitochondria, and mitochondria-bound BNIP3/BNIP3L can interact with LC3 to form the mitochondria-BNIP3/BNIP3L-LC3 autophagosome complex, inducing mitophagy 100. BNIP3/BNIP3L was transcriptionally regulated by NFKB or FOXO3/FOXO3A, linking mitophagy to fundamental signaling pathways 101,102. BNIP3/BNIP3L interacted with SPATA18 to clear ROS 103, and interacted with CDH6 to regulate mitophagy, maintaining mitochondrial homeostasis 104. Overexpression of BNIP3 improved ATP production in Parkinson's disease 105, and BNIP3/BNIP3L-mediated mitophagy protected against brain ischemic injury 106,107. These studies indicated that BNIP3/BNIP3L-mediated mitophagy played an important protective role in central nervous system diseases.

Our sequencing results indicated that the BNIP3 gene began to show downregulation on the first day after SCI, and was significantly downregulated on the third, fifth, and seventh days, suggesting a potential correlation between the downregulation of BNIP3 and the inhibition of mitophagy. To our surprise, in the cell model, we detected the inhibition of mitophagy simultaneously accompanied with the downregulation of BNIP3, indicating that we successfully simulated the downregulation of BNIP3 and the inhibition of mitophagy in the acute and subacute phase of SCI *in vitro*. Furthermore, during the reversal of oxidative stress-mediated inhibition of mitophagy by LIG, we also observed an upregulation of BNIP3, indicating that the enhanced effect of LIG on mitophagy is achieved through the upregulation of BNIP3. Building on this, the addition of siBNIP3 partially counteracted the enhanced effect of LIG on mitophagy, the state of which was still higher than that in the TBHP group, indicating that LIG-enhanced mitophagy may involve other mechanisms, with BNIP3 possibly being one of the most important pathways.

The strategies for repairing SCI mainly focus on two aspects: alleviating secondary damage, promoting neural plasticity and axonal regeneration 108. Oxidative stress reactions lead to a series of pathological damages and have become the focus of treatment. Post-SCI antioxidant strategies mainly include reducing ROS production, inhibiting LP, and scavenging free radicals 2,109. In recent years, traditional Chinese medicine has attracted considerable attention in the treatment of SCI and is considered a promising therapeutic approach. Several Chinese herbal extracts, including quercetin, resveratrol, and curcumin, have shown their ability to act as antioxidants and their effectiveness in treating SCI 110-115. The present study indicated that intraperitoneal injection of LIG significantly enhanced the ability to scavenge free radicals and reduce LP after SCI. The acute necrosis and secondary pathophysiological cascade reaction after SCI can trigger long-term apoptosis of neurons and neurodegeneration 116,117. Many drug interventions target upstream mitochondrial damage, such as free radical production and neuronal Ca^2+^ overload, to prevent apoptosis 118. In addition to inhibiting oxidative stress reactions, LIG also demonstrated its ability to prevent neuronal apoptosis through enhancing mitophagy in the present study.

Due to the alleviation of secondary pathological damage in SCI, LIG further promoted tissue and functional recovery in SCI mice. Axonal regeneration and synaptic formation are crucial for the conduction of neural signals and spinal cord function recovery. During axonal growth, mitochondria aggregate along the axon and determine axonal branching 119 implying their important roles. Following SCI, not only is mitochondrial function affected, but mitochondrial transport is also impaired 120. Transporting healthy mitochondria to the site of injury has a positive effect on axonal regeneration 121-123. Therefore, Mitochondrial quality control and homeostasis for producing more healthy mitochondria appear to be crucial 124. Mitophagy, as a mechanism for mitochondrial self-renewal, functions to maintain mitochondrial quality control and homeostasis 32-34. In this study, LIG enhanced mitophagy, indicating its potential role in mitochondrial quality, accompanied by continuous recovery of neuronal function, manifested by a gradual increase in the number of Nissl bodies. Traditional H&E staining showed ongoing repair of tissue defects, while immunofluorescence further confirmed the gradual increase in NF200 fluorescence signals in the repairing tissue area, indicating axonal regeneration. Based on the inhibition of pathological damages and the repair of tissue defects, LIG further promoted the recovery of spinal cord motor function. In MEP analysis, the increased latency and amplitude of the P wave indicated increased depolarization of spinal motor neurons or peripheral nerve motor fibers. Footprint analysis and BMS scores showed significant recovery of hind limb function in SCI mice.

However, this study also has certain limitations, as the intraperitoneal injection method of drug administration is too diffuse, lacks specificity, and is prone to false positives. Combining biological materials and targeted drug delivery systems to improve the local efficacy of LIG in SCI holds the potential to further enhance its effects. The results of this study have already suggested that LIG's effects on mitophagy and cell apoptosis may involve other pathways. In-depth research into these potential mechanisms can provide a more profound and comprehensive elucidation of the scientific issues addressed in this study and offer more scientific and effective strategies for repairing SCI.

## Conclusions

In summary, this study elucidated the state of mitophagy inhibition following SCI and its potential mechanisms, and confirmed the effects of LIG-enhanced mitophagy through BNIP3-LC3, providing new therapeutic targets and strategies for repairing SCI.

## Supplementary Material

Supplementary figures and tables.

## Figures and Tables

**Figure 9:**
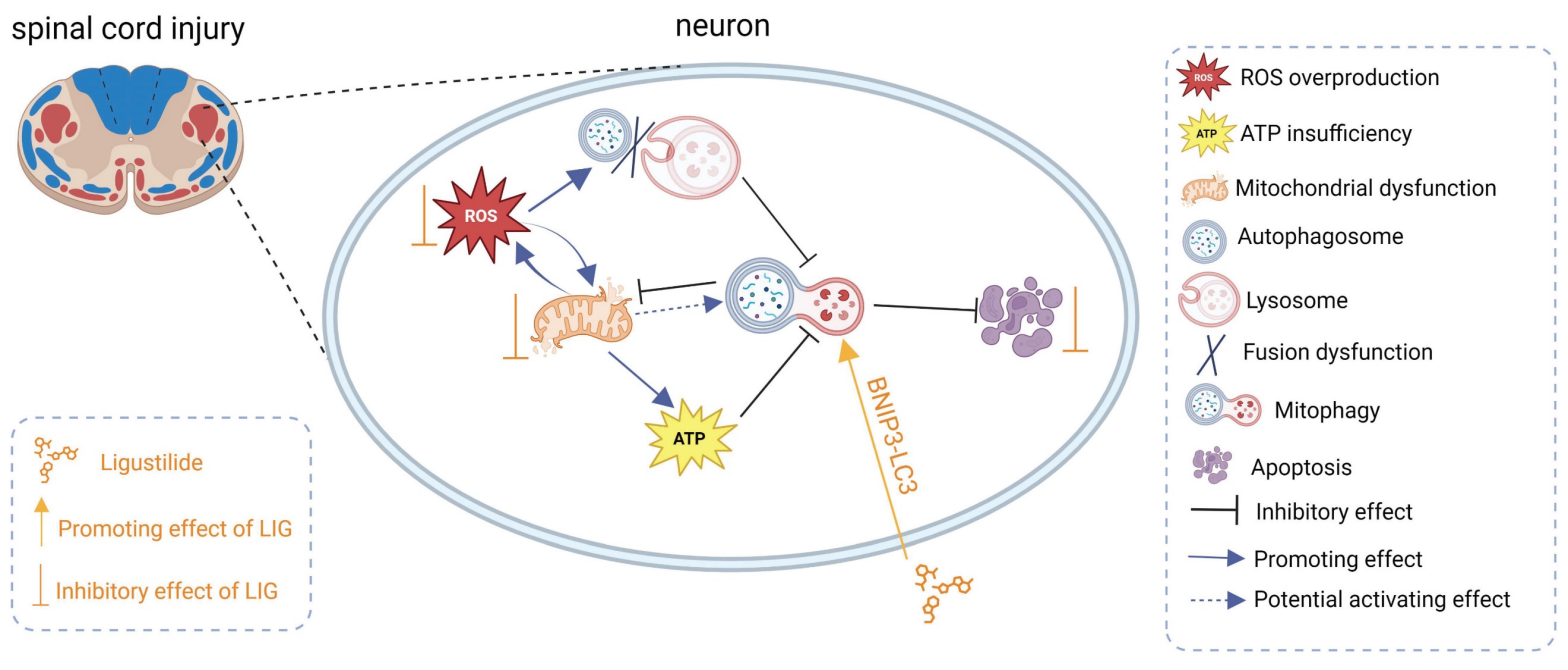
Schematic illustrating the underlying mechanisms of ROS-induced mitophagy inhibition and neuronal apoptosis, as well as the effects and mechanisms of LIG.

## Abbreviations

BNIP3: bcl2 interacting protein 3
BNIP3L: bcl2 interacting protein 3-like
Cyto C: Cytochrome C
DEGs: differentially expressed genes
DMSO: dimethyl sulfoxide
DRP-1: dynamin-related protein 1
DHE: dihydroethidium
GO: gene ontology
GSH: glutathione
KEGG: kyoto encyclopedia of genes and genomes
LIG: ligustilide
LP: lipid peroxidation
MEP: motor evoked potential
MDA: malondialdehyde
MMP: mitochondrial membrane potential
OD: optical density
PARP: poly ADP-ribose polymerase
PI: propidium iodide
PRKN: parkin RBR E3 ubiquitin protein ligase
ROS: reactive oxygen species
SCI: spinal cord injury
SOD: superoxide dismutase
TBHP: tert-butyl hydroperoxide
UA: Urolithin A
