# Supplementary material for: Enhancing mitophagy by ligustilide through BNIP3-LC3 interaction attenuates oxidative stress-induced neuronal apoptosis in spinal cord injury
Source: Int J Biol Sci. 2024 Aug 12;20(11):4382–406. doi: 10.7150/ijbs.98051 (PMC11379069; doi:10.7150/ijbs.98051)
Supplement: Supplementary file 1 — Supplementary figures and tables. [file ijbsv20p4382s1.pdf]

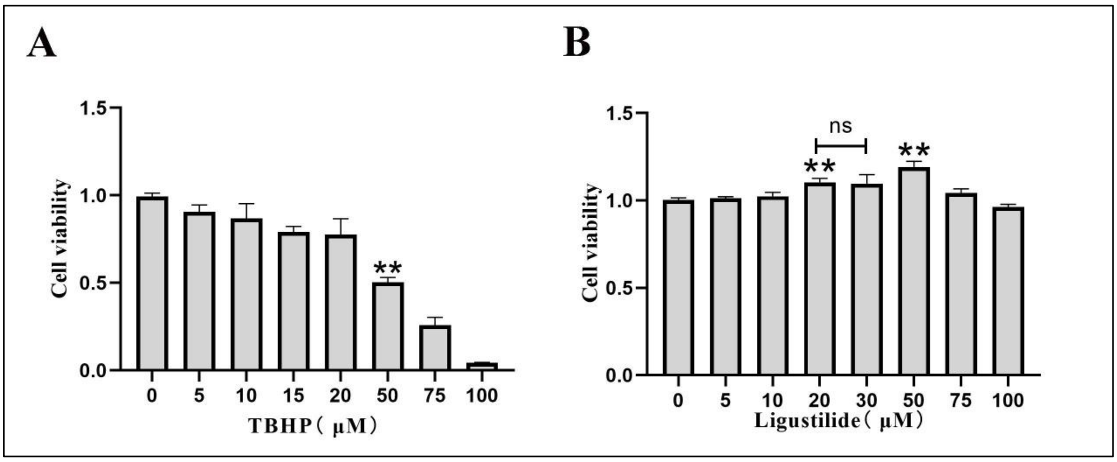

Figure S1. (**A, B**) Effects of varying doses of TBHP and ligustilide on the viability of PC12 cells (n=3). *p < 0.05 and **p < 0.01 versus control group, ns: not significant.

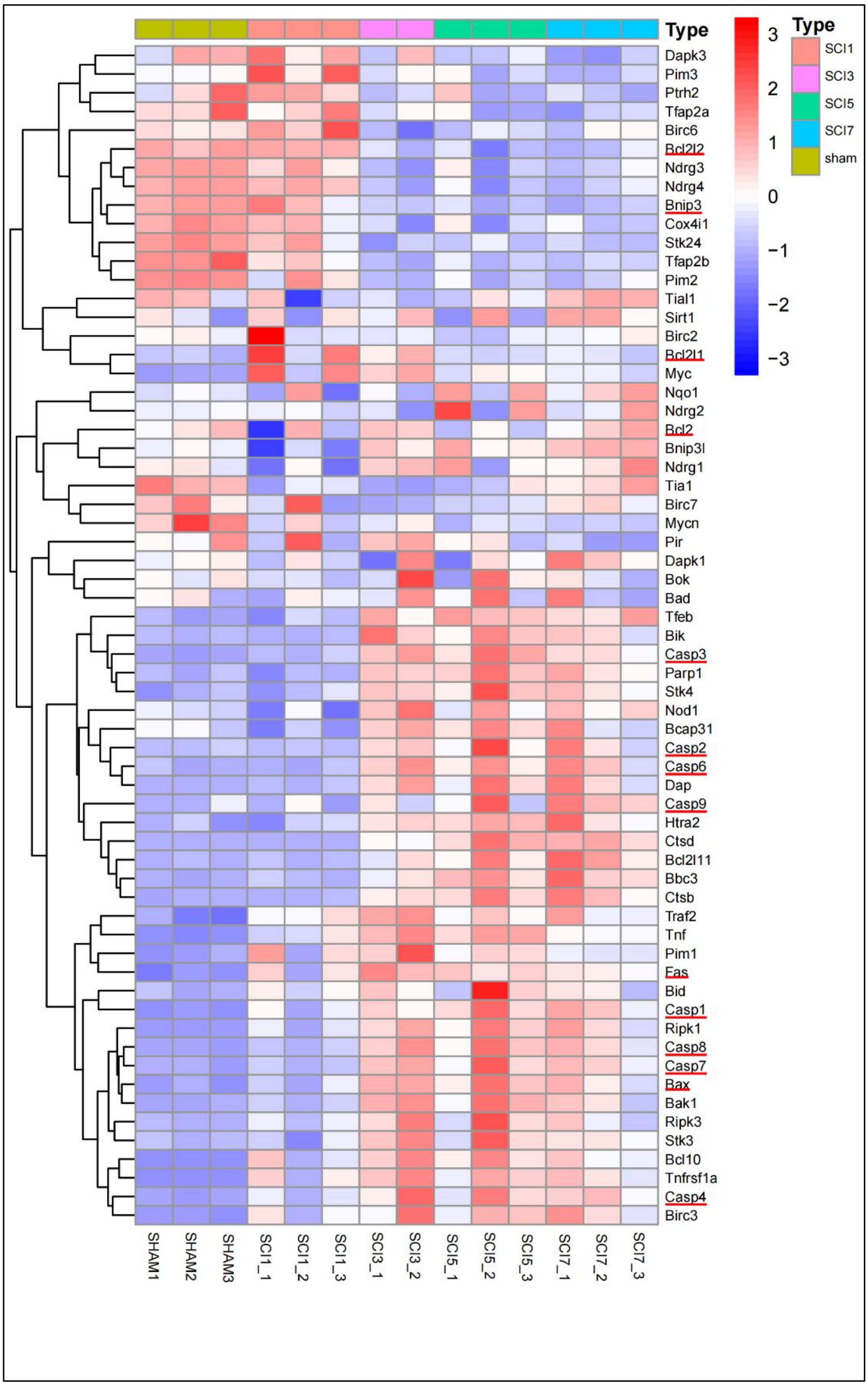

Figure S2. Heatmap displays the changes of genes related to apoptosis after 1, 3, 5, and 7 days following SCI. The typical genes of interest were marked with a red underline (n=3 animals for sham and SCI1,5,7d groups and n=2 animals for SCI3d group).

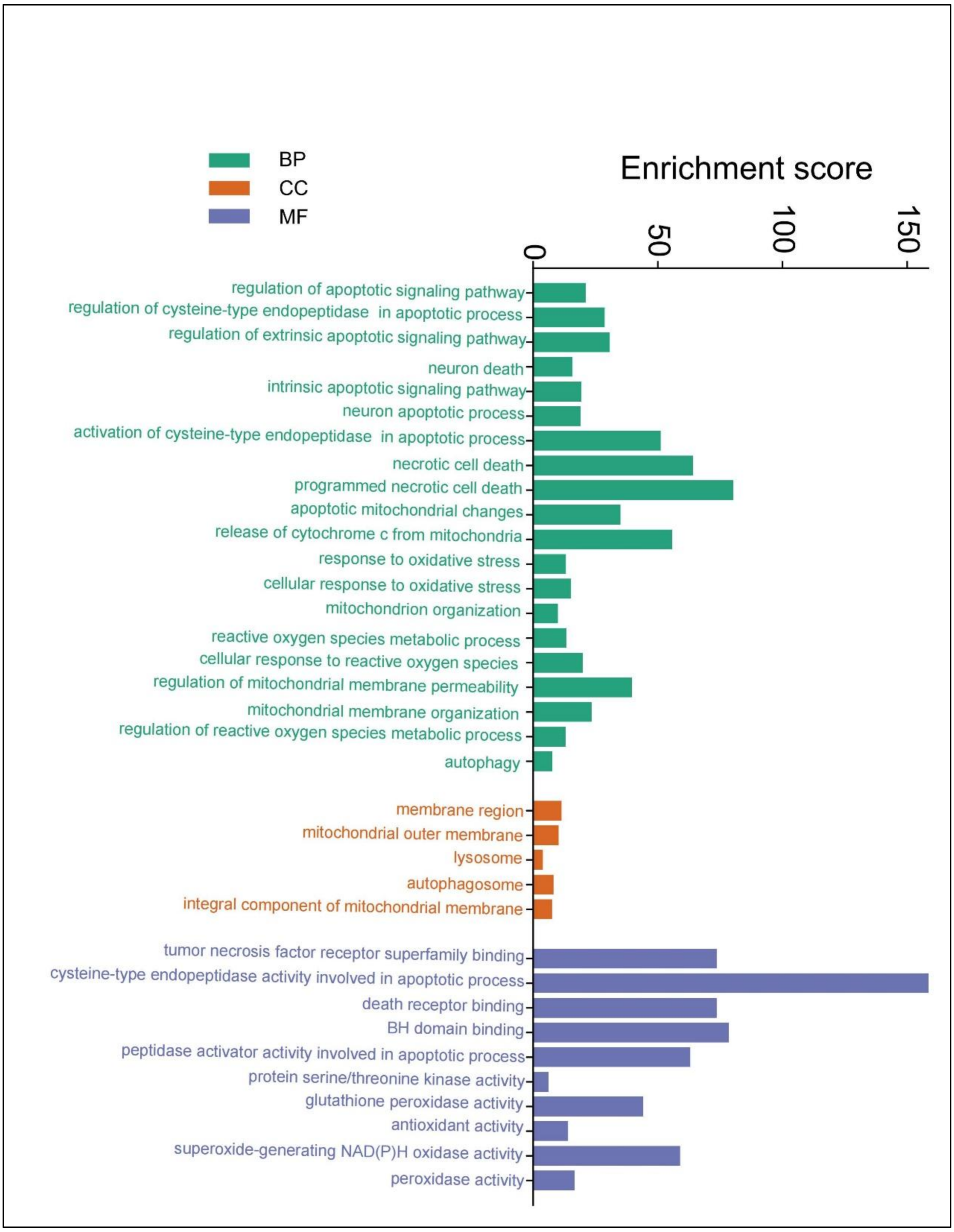

Figure S3. Gene Ontology (GO) enrichment analysis of DEGs between samples of sham and SCI5d (n=3 animals per group). BP (Biological Process), MF (Molecular Function) and CC (Cellular Component) were displayed in different colors separately.

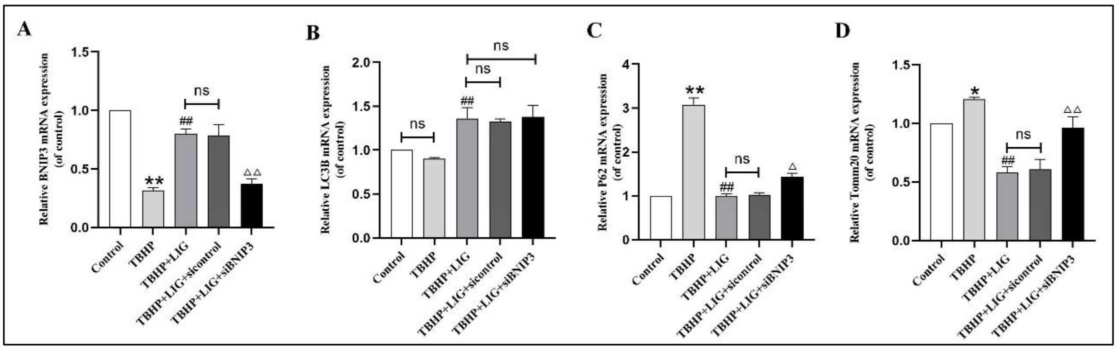

Figure S4. (**A-D**) Relative mRNA levels of LC3B, P62, Tomm20 and BNIP3 in Control, TBHP, TBHP+LIG, TBHP+LIG+siControl and TBHP+LIG+siBNIP3 groups (n=3). *$p < 0.05$ and **$p < 0.01$ versus the control group, $^{\#}p < 0.05$ and $^{\#\#}p < 0.01$ versus with the TBHP group, $^{\triangle}p < 0.05$ and $^{\triangle\triangle}p < 0.01$ versus the TBHP+LIG group, ns: not significant.

Table S1. Three siBNIP3 sequences and the control sequence.

siRNA	Sequences (5'-3')
Rat-siBNIP3-1F	GGACGAAGCAGCUCCAAGAGCTT
Rat-siBNIP3-1R	GCUCUUGGAGCUGCUUCGUCCTT
Rat-siBNIP3-2F	GAUUAUAUUGAGAGAAGAAGATT
Rat-siBNIP3-2R	UCUUCUUCUCUCAAUAUAAUCTT
Rat-siBNIP3-3F	GUUACUGUCUCAUCUGUUAGCTT
Rat-siBNIP3-3R	GCUAACAGAUGAGACAGUAACTT
Rat-siControl-F	UUCUCCGAACGUGUCACGUTT
Rat-siControl-R	ACGUGACACGUUCGGAGAATT

Table S2. The PCR primers used in the present study.

Primer names (RAT)	Sequences（5' to 3'）
RAT-MAP1LC3B-F	GAAGACCTTCAAACAGCGCC
RAT-MAP1LC3B-R	TGCCTTGGTAGGGGCTTAAC
RAT-SQSTM1-F	CTGAGTCGGGCATCGAGGTT
RAT-SQSTM1-R	TCTGGTGGGAGATGTGGGTA
RAT-Tomm20-F	CTTCCACCACCAGTGTTCCA
RAT-Tomm20-R	TCATGTTGGTGTCTGGCTCA
RAT-LAMP2-F	GAAAATGCCACTTGCCTTTATGC
RAT-LAMP2-R	AGGAAAAGCCAGGTCCGAAC
RAT-Bcl-2-F	GGTGAACTGGGGGAGGATTG
RAT-Bcl-2-R	AGAGCGATGTTGTCCACCAG
RAT-BAX-F	CACGTCTGCGGGGAGTCAC
RAT-BAX-R	TAGAAAAGGGCAACCACCCG
RAT-BNIP3-F	TTTAAACACCCGAAGCGCAC
RAT-BNIP3-R	AGTGGAAGTTGTCAGACGCC
RAT-Beclin1-F	GAATGGAGGGGTCTAAGGCG
RAT-Beclin1-R	CGTGTCCAGTTTCAGAGGCT
RAT-ULK1-F	TACACACCCTCTCCCCAAGT
RAT-ULK1-R	GGGACGAACGACATGGAAGT
RAT-ATG3-F	GCAGTTTTTGACTCCCTGGC
RAT-ATG3-R	GTGATCTCCAGCTGCCACAA
RAT-GAPDH-F	TGATGGGTGTGAACCACGAG
RAT-GAPDH-R	CCCATAACCCCCACAACACT
